# Proteomic and functional analysis identifies galectin-1 as a novel regulatory component of the cytotoxic granule machinery

**DOI:** 10.1038/cddis.2017.506

**Published:** 2017-12-07

**Authors:** Tiago Clemente, Narcisio J Vieira, Juan P Cerliani, Colin Adrain, Alexander Luthi, Mariana R Dominguez, Monica Yon, Fernanda C Barrence, Thalita B Riul, Richard D Cummings, Telma M Zorn, Sebastian Amigorena, Marcelo Dias-Baruffi, Maurício M Rodrigues, Seamus J Martin, Gabriel A Rabinovich, Gustavo P Amarante-Mendes

**Affiliations:** 1 Instituto de Ciências Biomédicas, Universidade de São Paulo, São Paulo, Brazil; 2 Instituto de Investigação em Imunologia, Instituto Nacional de Ciência e Tecnologia (INCT), São Paulo, Brazil; 3 Laboratorio de Inmunopatología, Instituto de Biología y Medicina Experimental (IBYME), Consejo Nacional de Investigaciones Científicas y Técnicas (CONICET), C1428 Buenos Aires, Argentina; 4 Instituto Gulbenkian de Ciência, Oeiras, Portugal; 5 Department of Genetics, Smurfit Institute, Trinity College, Dublin, Ireland; 6 Centro de Terapia Celular e Molecular (CTCMol) and Departamento de Microbiologia, Imunologia e Parasitologia, Universidade Federal de São Paulo—Escola Paulista de Medicina, São Paulo, São Paulo, Brazil; 7 Departamento de Biologia Celular e do Desenvolvimento, Instituto de Ciências Biomédicas, Universidade de São Paulo, São Paulo, Brazil; 8 Departamento de Análises Clínicas, Toxicológicas e Bromatológicas, Faculdade de Ciências Farmacêuticas de Ribeirão Preto, Universidade de São Paulo, Ribeirão Preto, Brazil; 9 Department of Surgery, Beth Israel Deaconess Medical Center, Harvard Medical School, Boston, MA, USA; 10 Institut Curie, PSL Research University, INSERM U932, Paris Cedex 05, France; 11 Departamento de Química Biológica, Facultad de Ciencias Exactas y Naturales, Universidad de Buenos Aires, C1428 Buenos Aires, Argentina

## Abstract

Secretory granules released by cytotoxic T lymphocytes (CTLs) are powerful weapons against intracellular microbes and tumor cells. Despite significant progress, there is still limited information on the molecular mechanisms implicated in target-driven degranulation, effector cell survival and composition and structure of the lytic granules. Here, using a proteomic approach we identified a panel of putative cytotoxic granule proteins, including some already known granule constituents and novel proteins that contribute to regulate the CTL lytic machinery. Particularly, we identified galectin-1 (Gal1), an endogenous immune regulatory lectin, as an integral component of the secretory granule machinery and unveil the unexpected function of this lectin in regulating CTL killing activity. Mechanistic studies revealed the ability of Gal1 to control the non-secretory lytic pathway by influencing Fas–Fas ligand interactions. This study offers new insights on the composition of the cytotoxic granule machinery, highlighting the dynamic cross talk between secretory and non-secretory pathways in controlling CTL lytic function.

Immune-mediated elimination of intracellular microbes and cancer cells is accomplished by a complex lytic process orchestrated by CD8^+^ cytotoxic T lymphocytes (CTLs) and NK cells.^1, 2, 3^ In response to antigen-specific recognition, biochemically defined signaling events triggered by T-cell receptor (TCR)/CD3 engagement and crosslinking of accessory molecules, leads to CD8 T-cell activation and differentiation. Subsequent TCR/CD3 stimulation results in release of a fraction of cytotoxic granules at the focal point of CTL/target cell contact, ensuring that a single CTL can kill multiple target cells in a specific fashion avoiding ‘off-target’ cytotoxic effects.^1, 2, 3^

Among relevant molecules that serve as effectors of CTL-mediated cytotoxicity, perforin, granzyme A and granzyme B have been shown to have major roles in killing pathogen-invaded as well cancer cells.^4, 5, 6, 7^ Current knowledge assumes that perforin delivers granzymes A and B, and most certainly other granule molecules, to the cytosolic compartment of target cells.^5, 7, 8^ In particular, granzyme B has been shown to be an efficient initiator of the apoptotic machinery,^9, 10^ whereas granzyme A may account for caspase-independent events associated with target cell death.^6^ However, mice deficient in any of these molecules, either alone or in combination, are still able to eradicate infected cells and some tumors,^11^ suggesting that alternative non-overlapping or partially overlapping pathways may have compensatory roles.

In addition to their pro-apoptotic effects, mediators released during the CTL attack may also function as immune regulators controlling the activity of other immune cells in tumor, infectious or inflammatory microenvironments. For example, *β*-chemokines, a CD8^+^ antiviral factor and many cytokines, including interferon (IFN)-*γ*, and tumor necrosis factor are released by activated CTLs.^12, 13^ In addition, regulatory mediators such as serpin PI-9 (SPI-6)^14^ and the lysosomal protein cathepsin B^15^ may contribute to protect CTLs and NK cells from their own lytic machinery. Because of the extreme complexity and incomplete understanding of the molecular mechanisms that control granule-mediated cytotoxic cell killing, identification of positive and negative regulators associated with the purified CTL/NK granule fraction may contribute to dissect the complexity of this lytic machinery with the ultimate goal of manipulating this pathway for therapeutic purposes.

Here we use a large-scale proteomic approach to identify novel cytotoxic granule proteins. Particularly, we identified galectin-1 (Gal1) as one of the most abundant CTL granule constituents and showed by confocal microscopy that Gal1 colocalizes with perforin, granzyme B, Lamp-1 and Lamp-2. In addition, electron microscopy images clearly indicate the presence of Gal1 at the border and inside CTL granules. Importantly, CTLs from *Lgals1*^*−/−*^ mice display altered capacity to eliminate antigen-specific targets *in vivo*. Mechanistic studies suggest that Gal1 can modulate cell surface Fas ligand retention and fine tune CTL activity. This study identifies an important new component of the cytotoxic granule machinery and reveals a different level of regulation of CTL lytic function.

## Results

### Proteomic analysis identifies major CTL granule proteins

**Figure 1:**
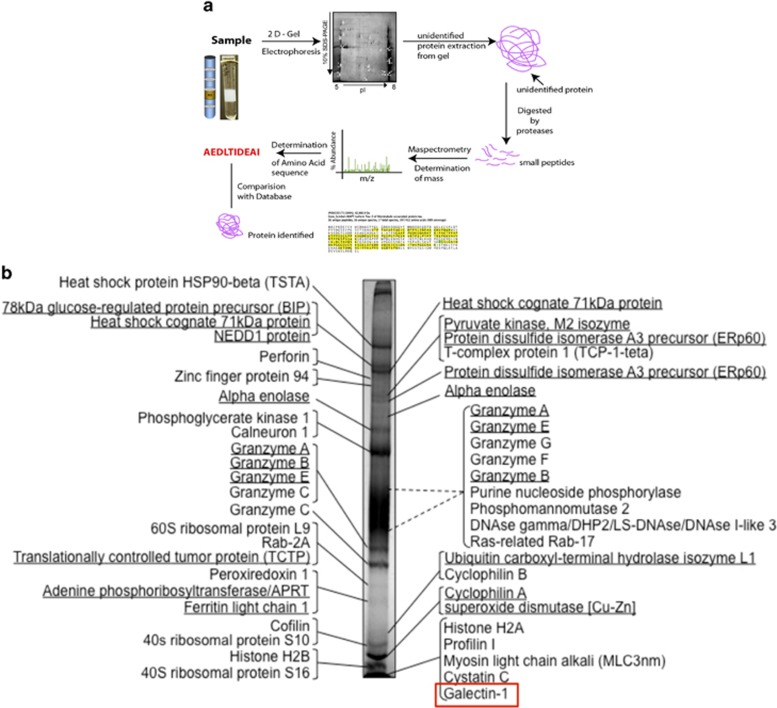
Proteomic analysis of cytotoxic cell granules. (**a**) Proteomic strategy; (**b**) 1D gel analysis of cytotoxic granules and main proteins detected by mass spectrometry

To identify novel mediators that control the CTL lytic machinery, cytoplasmic granules of mouse H-2^d^-specific cell line B6.1 were isolated as described.^16^ The major bands observed in Coomassie blue-stained 1D SDS-PAGE were sliced out of the gel and subjected to in-gel trypsin digestion and MS/MS analysis (Figure 1a). Because of the complexity of the samples, as each band most likely contains several different proteins, only proteins with high MS-Fit score, peptide coverage above 35% and a significant number of peptide matches were selected in this first scrutiny. Thirty-nine proteins were therefore nominated as the most abundant proteins in our CTL granule fraction (Table 1), including the expected perforin and the granzymes A, B, C, E, F and G. Although the MS/MS analysis did reveal the presence of granzymes D and H in our samples (data not shown), both of the scores for these granzymes did not reach our predetermined threshold as to be considered among the most abundant proteins in our preparation. The positions of all these proteins are shown in a Coomassie blue-stained 12% SDS-PAGE gel (Figure 1b). As a single band may be composed of one or two major proteins or several minor proteins, we decided not to select bands according to their abundance or intensity as it would have been incorrect to assign relative concentration of the selected proteins based on the MS-Fit score or any data from our MS/MS analysis, including size and numbers of matched peaks.

To maximize the identification of the proteins present in our CTL granule fraction, we separated samples by 2D SDS-PAGE, using different pI gradients (Supplementary Figure 1). As most of the granzymes are highly basic proteins and comprise, as far as we know, the most abundant constituents of CTL granules, it was not surprising to find out that a large amount of proteins were not resolved by pI 3-6 and pI 5-8 IEF gradients. In fact, a strong label was always detected at the very basic rim of pI 3-6 and 5-8 gels (Supplementary Figure 1). Although we ran many pI 7-10 2D SDS-PAGE gels, none provided us with a good resolution of the basic CTL granule proteins. Therefore, it was not possible to isolate single spots of the basic proteins. However, by consistently sampling the basic edge of both pI 3-6 and 5-8 gels, we successfully identified some of the granzymes (Table 2). In addition, proteomic analysis of spots isolated from 2D gels revealed some proteins also found in 1D gel, such as cyclophilin A, adenine phosphoribosyltransferase, translationally controlled tumor protein and heat-shock cognate 71 kDa protein (Table 2). Together, both strategies revealed a total of 246 proteins among which, we found proteins previously shown to be localized in lysosomes or cytotoxic granules as well as novel putative candidates (Supplementary Table I). Interestingly, mass spectrometry analysis revealed the presence of Gal1, a prototype *β*-galactoside-binding lectin, as a major protein of secretory granules (Figure 1b).

### Validation of mass spec data

**Figure 2:**
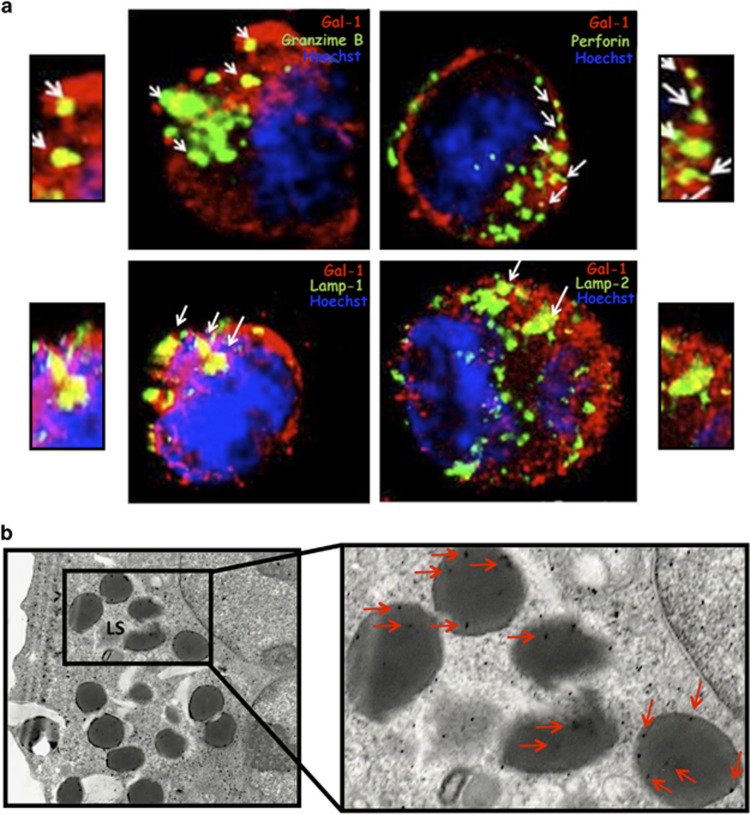
Confocal and electron microscopy of YT cells. (**a**) Confocal microscopy showing colocalization of Gal1 with granzyme B, perforin, Lamp-1 and Lamp-2; (**b**) electron microscopy showing Gal1 localization inside cytotoxic granules

To validate the mass spectrometry data, we first evaluated the expression of selected proteins in the NK cell line YT and compared to the expression found in other cell lines of non-cytotoxic cell origin. Western blot analysis shows that YT cells represent the only cell line that express granzyme B (A). Remarkably, all the other proteins were more abundant in YT cells compared to the other cell lines, particularly TRAIL, galectin-3 (Gal3) and DNAse *γ*. The expression of the selected proteins in YT cells was confirmed by immunofluorescence (Supplementary Figure 2B). In particular, confocal as well as electron microscopy revealed that Gal1 was present inside cytotoxic granules, and colocalized with granzyme B, perforin, Lamp-1 and Lamp-2 (Figure 2).

### *In vivo* cytotoxic assay revealed a role for endogenous Gal1 in killing specific CTL targets

**Figure 3:**
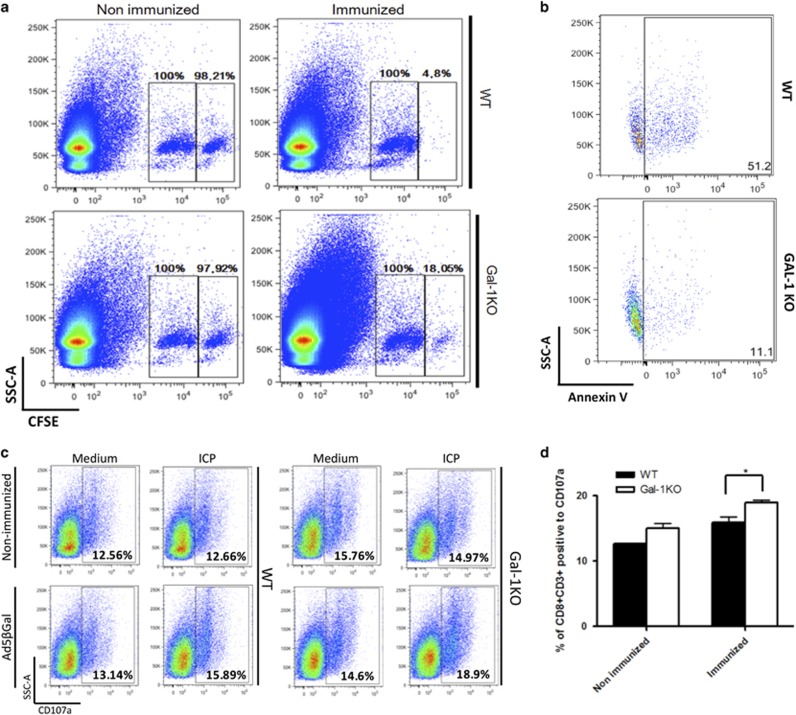
Altered cytotoxic activity of *Lgals1*^*−/−*^ CD8^+^ T cells. (**a**) *In vivo* antigen-specific target elimination by WT or *Lgals1*^*−/−*^ C57BL/6 mice immunized or not with 2 × 10^8^ PFU of Ad5*β*-gal 8 days before. (**b**) *In vitro* killing of L1210 targets by CD8^**+**^ T cells obtained from the peritoneal cavity of WT or *Lgals1*^*−/−*^ C57BL/6 mice injected 12 days before with L1210 cells. (**c** and **d**) WT or *Lgals1*^*−/−*^CD8^**+**^ T-cell degranulation upon antigen-specific stimulation, as measured by Lamp-1 (CD107a) externalization

**Figure 4:**
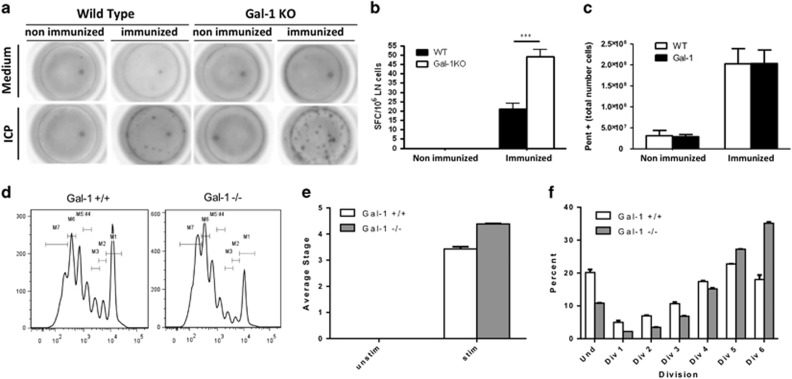
*Lgals1*^*−/−*^ CD8^+^ T cells displayed enhanced *in vitro* and *in vivo* proliferating capacity compared to WT cells. (**a** and **b**) ELISPOT analysis of IFN-*γ*-producing lymph node cells obtained from WT or *Lgals1*^*−/−*^ C57Bl/6 mice immunized or not with Ad5.*β*gal for 7 days and *in vitro* stimulated with cognate peptide ICP. (**c**) Antigen-specific CD8^**+**^ T cells obtained from spleens of WT or *Lgals1*^*−/−*^ C57Bl/6 mice immunized or not with Ad5.*β*gal for 7 days, assessed as pentamer-positive events. (**d**–**f**) *In vitro* proliferation of WT or *Lgals1*^*−/−*^ CD8 T cells stimulated for 72 h with 40 U/ml IL-2 and 10 *μ*l/ml of CD3/CD28 beads. (**d**) Histogram represents the peaks of CFSE dilution of dividing cell populations. Bars represent (**e**) average stage of division and (**f**) percentage of cells in different stages of divisions in response to stimulation by IL-2 and anti-CD3/anti-CD28 mAb as determined from the CFSE dilution profile in **d**

The relative abundance of Gal1 in secretory granules prompted us to evaluate its role as a component of the cytotoxic cell machinery. We used an *in vivo* CTL assay well established in our laboratory^17^ based on recombinant adenovirus vaccination to elicit specific CD8 T-lymphocyte activation and expansion. In this cytotoxic *in vivo* assay, target elimination is completely dependent on CD8^+^ T cells (Supplementary Figure 3). Importantly, elimination of CTL-specific targets was impaired in Gal1-deficient (*Lgals1*^*−/−*^) mice, suggesting an *in vivo* role for this lectin in CTL killing (Figure 3a). Similar results were obtained *in vitro* using CD8^+^ T cells isolated from wild-type (WT) or *Lgals1*^*−/−*^ mice i.p. injected 12 days earlier with L1210 cells. Whereas CD8^+^ WT T cells killed around 50% of L1210 targets *in vitro*, *Lgals1*^*−/−*^ CD8^+^ T cells eliminated around 10% of the same targets (Figure 3b). This altered lytic capacity was not due to impaired degranulation as *Lgals1*^*−/−*^ CD8^+^ T lymphocytes degranulated as much as WT CD8^+^ T cells, as measured by CD107a externalization upon antigenic stimulation (Figures 3c and d). Moreover, reduced CTL killing observed in *Lgals1*^*−/−*^ mice was not the result of a deficiency in specific CD8+ T-lymphocyte expansion. On the contrary, ELISPOT analysis revealed a higher frequency of IFN-*γ*-producing lymph node CD8^+^ T cells in *Lgals1*^*−/−*^ compared to WT C57BL/6 mice, in response to cognate peptide (Figures 4a and b). In addition, the numbers of pentamer-positive CD8^+^ T cells were similar in WT and *Lgals1*^*−/−*^ mice following 7 days of immunization with Ad5.*β*-gal (Figure 4c). Finally, CSFE staining of CD8^+^ T cells obtaining from WT or *Lgals1*^*−/−*^ stimulated *in vitro* for 72 h with IL-2 and CD3/CD28 beads also revealed increased proliferation of *Lgals1*^*−/−*^ cells (Figures 4d–f). Taken together, these data suggest that although *Lgals1*^*−/−*^ CTLs are expanded at higher frequency and are capable of degranulating properly upon specific stimulation, these cells experience limitations in killing specific targets.

### CTLs lacking Gal1 show altered Fas-mediated cytotoxicity

**Figure 5:**
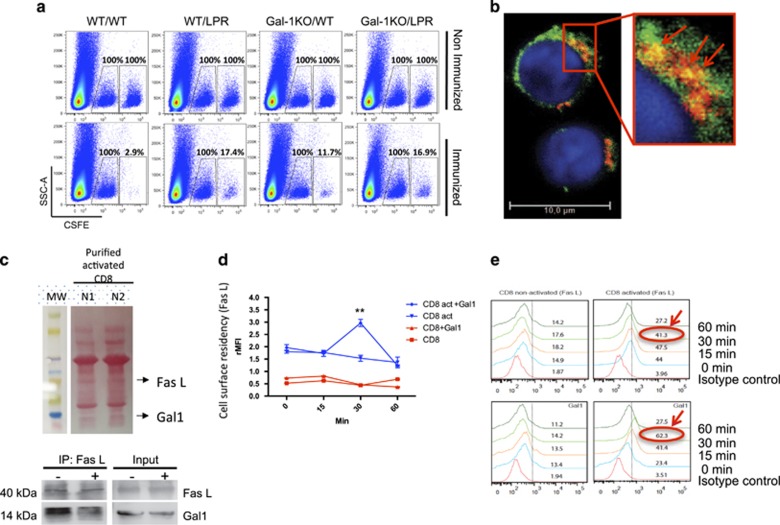
*Lgals1*^*−/−*^ CD8+ T cells are as efficient as WT CD8+ T cells at killing peptide-pulsed FAS-deficient target cells. (**a**) *In vivo* cytotoxic assay comparing the efficiency of WT or *Lgals1*^*−/−*^ mice to eliminate Fas-sufficient or -deficient target cells. (**b**) Colocalization of FasL and Gal1 in YT cells by confocal microscopy. (**c**) Co-immunoprecipitation followed by immunoblotting of Gal1 and FasL expression in lysates from CD8 purified cells incubated with anti-CD3 and anti-CD28 mAb for 18 h and further stimulated (+) or not (−) with recombinant Gal1 (rGal1). Input, whole cell lysate; IP, immunoprecipitation. Ponceau S staining of all the proteins ran is shown (upper panel). (**d** and **e**) Flow cytometry analysis of FasL expression in non-permeabilized, purified CD8^+^ T cells incubated or not with anti-CD3/anti-CD28 mAb for 18 h and then stimulated with PBS or rGal1 for the indicated time periods. Nonspecific binding determined with isotype-matched control antibodies is shown. (**d**) Curves of rMFI (median fluorescence intensity of specific marker signal–median fluorescence intensity of unspecific signal) for each time period analyzed. (**e**) Retention of FasL on the surface of purified CD8^+^ T cells. Numbers show the percentage of positive cells

*In vivo* CTL killing operates largely through perforin and granzymes via the granule exocytosis pathway, although the death receptor pathway, mainly mediated by Fas/FasL interaction also has an important role.^18, 19^ Using WT (Fas-sufficient) and *lpr* (Fas-deficient) targets we confirmed this observation. Whereas more than 95% of Fas-sufficient targets were eliminated, only around 80% of FAS-deficient *lpr* targets were eliminated in WT immunized mice (Figure 5a). Importantly, immunized *Lgals1*^*−/−*^ mice eliminated equally (less efficiently) Fas-sufficient and Fas-deficient targets (around 80–85% Figure 5a). These results show that the deficiency observed in *Lgals1*^*−/−*^ CTL killing recapitulates killing defects of Fas-deficient targets by WT mice, suggesting that in the absence of Gal1, FAS/FASL pathway seems to be inoperative.

### Gal1 acts by retaining FasL on the CTL surface and preventing its internalization

To explore the mechanisms by which Gal1–glycan interactions control CTL cytotoxicity, we performed *in vitro* functional assays. Because FasL (CD95 ligand) is a 40 kDa type II membrane protein composed of three potential *N*-glycosylation sites (Asn^184^, Asn^250^ and Asn^260^,^20, 21^ we hypothesized that Gal1–glycan interactions may modulate Fas–FasL interactions by specifically retaining FasL on the surface of CTLs, thereby prolonging interactions with Fas-positive target cells. Co-immunoprecipitation experiments with lysates of activated CD8^+^ T cells treated with recombinant Gal1 revealed specific interactions between Gal1 and FasL (Figure 5c). Notably, flow cytometric analysis of non-permeabilized cells demonstrated time-dependent retention of FasL on the surface of activated CTLs exposed to Gal1, an effect that was more pronounced at 30 min of incubation, compared to cells treated with vehicle control (Figures 5d and e), suggesting that Gal1 may contribute to prolong Fas–FasL interactions at the CTL–target interface. Thus, association of Gal1 to FasL promotes retention of this glycoprotein on the surface of CTLs.

## Discussion

Exocytosis of lytic granules by cytotoxic cells is a Ca^2+^-dependent phenomenon that occurs in a very coordinated manner, initiated by the triggering of the TCR and culminating in target-directed release of their constituents.^2^ To fully understand the molecular basis of CTL killing individual exploration of the biochemical and functional nature of purified cytotoxic granules is mandatory. Although some of the molecules are directly responsible for killing target cells, many others may be associated with positive or negative immune regulatory activities.^5, 13^

The data presented here comprise a comprehensive list of lytic granules components. Although our strategy is far from revealing the totality of proteins present in the granules of cytotoxic cells, it definitely disclosed key components of these highly specialized organelles. In this regard, it is important to keep in mind that MS/MS identification of complex protein mixtures is usually biased toward the most abundant proteins, as clearly shown in the case of proteomic analysis of the *Arabidopsis thaliana* chloroplast proteome.^22^ One of the reasons is that, although genomic approaches can use protocols designed to increase the number of copies of genes of interest, proteomic technology is still restrained by the fact that there is no available methodology to amplify proteins in any biological sample. Besides, not every protein in a given sample can be properly distinguished from others by any available methodology.

We used a combination of one- and two-dimension SDS-PAGE to maximize our chances to identify the cytotoxic granule components present on the cytoplasmic compartment of CTLs. In one hand, one-dimension analysis provided us with a picture of the most abundant proteins, based on the molecular weight of the major bands in Coomassie blue-stained gels. It is reasonable to speculate that individual bands analyzed may be composed of a number of proteins with similar molecular weight. Many of these proteins are probably concealed by the most abundant ones and, therefore, cannot be recognized in our scrutiny. On the other hand, although two-dimension analysis generates isolated spots that most likely contain a single protein, basic or acidic molecules as well as high-molecular-weight proteins are not properly resolved in 2D gels. In addition, technical difficulties limit isolation of any given organelle to (the extent of 100%) purity (homogeneity).

However, despite the intrinsic obstacles to unravel the composition of the cytotoxic granules, we have successfully identified novel granule-associated proteins as well as others already known to be present in either CTL or NK granules. Importantly, they were all found to be associated with the organelle fraction enriched for many of the proteins known to be present in the lytic granules. Moreover, all received a high-confidence MS-Fit score and displayed significant peptide coverage.

Two major strategies have been used to assign proteins to specific subcellular compartments. The first uses confocal microscopy approaches to reveal colocalization with well-established markers. The sensitivity of this method may not allow proper labeling of proteins present at low concentration. Furthermore, if the protein is also present in any other compartment, the specific signal may be disguised. That might well be the case of a fraction of the proteins identified in this study. The second approach is based on the ectopic expression of constructs designed to contain molecular tags. Although this strategy greatly increases the detection limit for low abundance proteins, it is possible that proteins expressed in such way may not be properly folded and/or transported within the cell and, consequently, ended up in different compartment than the endogenous counterparts. Nevertheless, it is important to emphasize that the novel proteins presented in this study may not have an exclusive cytotoxic granule localization, as is clearly the case of Gal1, an immune regulatory lectin widely distributed in multiple cells, tissues and subcellular compartments.

Gal1 is a prototype member of a family of endogenous lectins, which functions intracellularly by interacting with intracellular signaling components or extracellularly by recognizing *N*-acetyllactosamine (Gal*β*^1, 2, 3, 4^-GlcNAc; LacNAc) residues on a myriad of cell surface glycosylated receptors, including CD45, CD43 and CD7.^23^ This lectin has been implicated in the regulation of innate and adaptive immune responses through multiple mechanisms. In the periphery, Gal1 selectively dampens Th1 and Th17 responses,^24, 25^ induces IL-10 secretion,^26, 27^ inhibits T-cell trafficking^28^ and decreases antigen-presenting capacity by macrophages.^24^ Furthermore, exposure to Gal1 promotes the differentiation of IL-27-producing tolerogenic dendritic cells^29^ and favors the expansion of inducible T regulatory cells.^30^ Within the CD8^+^ T-cell compartment, Gal1 is induced in activated CD8 T cells and functions as an autocrine negative regulator of CD8^+^ T-cell binding, signal transduction and burst size.^31^ Gal1 deficiency results in increased frequency of CD8^+^ T cells and CD8-mediated tumor rejection in several tumor models.^32, 33, 34^ Here we defined a novel role for Gal1 as a component of CTL lytic granules that contributes to CTL effector function. Given the complexity of CTL biology, the overall positive or negative effects of this lectin in CTL function might depend on the pathophysiologic context and the relative contribution of this lectin to different stages of CTL lifespan, including activation, expansion, granule release and contraction. In this regard, we found here that Gal1 negatively controls proliferation of CD8^+^ T cells, whereas amplifies FasL-mediated killing activity. Moreover, our findings show that Gal1 may retain FasL on the surface of CTLs, suggesting that this lectin could delay FasL endocytosis and prolong interactions with Fas-positive target cells. Interestingly, this lectin has been shown to increase cell surface residency and prevent endocytosis of various glycosylated receptors, including the CD45 phosphatase in microglia cells^23^ and vascular endothelial growth factor receptor 2 on the surface of endothelial cells.^32^ Thus, lectin–glycan interactions can amplify or interrupt cell–cell communication by modulating endocytosis, trafficking and signaling of canonical receptors. Importantly, as blockade of Gal1 in most cancer models results in CD8^+^ T-cell-mediated tumor rejection,^32, 33^ it seems apparent that under conditions in which Gal1 secretion is substantially increased (i.e., tumor or inflammatory microenvironments), contraction of the CD8^+^ T-cell compartment and inhibition of CTL function will certainly prevail.

In conclusion, using an interdisciplinary approach ranging from proteomics search to biochemical and functional assays, we identified a novel role of Gal1 as an integral component of the CTL lytic machinery. Moreover, our proteomics strategy sets the stage for further studies to unravel the function of the newly identified proteins in cytotoxic cell degranulation, killing and immunomodulation.

## Materials and methods

### Mice and ethics statement

Six- to eight-week-old WT, CD8^*−/−*^ or Gal1-deficient (*Lgals1*^*−/−*^) C57BL/6 mice (all males) were housed at our animal facility at the Institute of Biomedical Sciences, University of Sao Paulo (ICB-USP). This study was carried out in strict accordance with the recommendations in the Guide for the Care and Use of Laboratory Animals of the Brazilian National Council of Animal Experimentation (http://www.cobea.org.br/). The protocols were approved by the Animal Ethics Committee of the ICB-USP.

### Cell culture

The L1210 and the NK line YT were maintained in RPMI-1640 supplemented with 10% FCS, 25 mM HEPES, 2 mM l-glutamine, 100 U/ml penicillin and 100 *μ*g/ml streptomycin.

### Antibodies and reagents

MytoTracker and LysoTracker were obtained from Molecular Probes (Eugene, OR, USA). Antibodies were from different sources. Anti-perforin (clone *δ*G9), anti-TRAIL (clone III6F) and anti-granzyme A mAbs (clone GA6) were purchased from Alexis Biochemicals/Enzo Life Sciences, Inc. (Farmingdale, NY, USA). Anti-Gal1 and anti-galectin-3 mAbs were obtained from R&D Systems, Inc. (Minneapolis, MN, USA). Anti-DNase *γ* mAb (Ab-1) was purchased from Oncogene Research Products (La Jolla, CA, USA) and anti-granzyme B pAb (ab4059) was from Abcam (Cambridge, UK). Anti-perforin mAb (clone CB5.4) was acquired from Apotech Co. (Epalinges, Switzerland). Anti-cofilin and anti-profilin pAbs were purchased from Cytoskeleton, Inc. (Denver, CO, USA). Secondary antibodies coupled with Alexa Red 546 or Alexa 488 were acquired from Invitrogen (Carlsbad, CA, EUA). CTL granule preparation was kindly provided by Dr. Jürg Tschopp and corresponds to formulation described in ref. 16.

### Separation of granule proteins by electrophoresis

CTL granule proteins were precipitated in 13% TCA. After 1 h incubation at 4 °C, samples were centrifuged at 15 000 × *g* for 15 min at 4 °C and pellets were washed twice in 1 ml of cold acetone. For 1D analysis, the dried pellets were resuspended in SDS-PAGE loading buffer (100 mM DTT, 2% SDS, 10% glycerol, 0,1% bromophenol blue and 50 mM Tris-Cl, pH 6.8) and separated by SDS-PAGE according to Laemmli.^35^ For 2D analysis, pellets were dissolved in IEF sample buffer (8 M urea, 4% CHAPS, 0.05% SDS, 100 mM DTT, 0.5 % Bio-Lyte ampholyte, plus a trace of bromophenol blue) and initially used to rehydrated IPG strips overnight. First dimension IEF was carried out on a Protean IEF Cell (Bio-Rad, Hercules, CA, USA) for a total of 55 000 Vh and second dimension on a Protean II xi Cell (Bio-Rad). After protein separation, gels were stained with either Coomassie brilliant blue or silver nitrate as described.^36^

### Mass spectrometry analysis

Spots were excised from silver-stained 2D gels, washed once in oxidation buffer (15 mM K_3_Fe(CN)_6_ and 50 mM Na_2_S_2_O_3_) and then successively in 50% methanol/40% acetic acid. After equilibration in NH_4_HCO_3_, gel pieces were dehydrated in 100% acetonitrile and desiccated completely in Speed-vac at low heat setting. In-gel tryptic digestion was performed overnight at 37 °C in 12 *μ*l of 10 *μ*g/ml trypsin in 25 mM NH_4_HCO_3_/0.1% *n*-octylglucoside. Peptide mass fingerprinting was performed by MALDI-MS in a Voyager-DE PRO Biospectrometry Workstation (Applied Biosystems, Foster City, CA, USA). Tryptic fragments were spotted onto the MALDI sample probe, in the presence of *α*-cyano-4-hydroxy-trans-cinnamic acid (Sigma, St Louis, MO, USA). Monoisotopic peptide masses were assigned and used to search the Swiss-Prot database using the MS-Fit program (University of California, San Francisco—http:/prospector.ucsf.edu/).

### Western blot analysis

Protein samples were resolved under reducing conditions for 2 h at 80 V in SDS-polyacrylamide gels, as previously described.^37^ Briefly, cells were collected, washed once in ice-cold PBS, lysed directly in SDS-PAGE loading buffer and boiled for 5 min before SDS-PAGE. Separated proteins were blotted onto nitrocellulose or PVDF membranes at 150 mA overnight. Blots were blocked in TBST (10 mM Tris-HCl, pH 7.4, 150 mM NaCl and 0.05% Tween) containing 0.1% sodium azide and 5% nonfat dried milk and then probed for 2 h with an appropriate dilution of the primary antibody. Reactions were detected with suitable secondary antibody conjugated to horseradish peroxidase (Amersham, Arlington, IL, USA) using enhanced chemiluminescence (Pierce, Rockford, IL, USA).

### Immunofluorescence and confocal microscopy

Colocalization of Gal1 and cytotoxic granule proteins were performed as described.^38^ In brief, cells were collected, washed with cold PBS, placed on poly-l-lysine (Sigma)-covered glass slides and incubated for 30 min at 37 °C. Supernatants were removed and cells fixed in 2% paraformaldehyde for another 30 min at 4 °C before subjected to permeabilization with PBS containing 0.1% Triton X-100 and 1% BSA. Cells were washed again and labeled with desired antibodies for 30 min at 37 °C before analyzed in a Zeiss LSM 510 microscope (Carl Zeiss Jena GmbH, Jena, Germany) using C-Apochromat × 40 and × 60 (zoom of × 120) objectives. The fluorophores used in all experiments were Alexa Red 546 (red image) or Alexa 488 (green Image). Pinhole size was 95.6 *μ*m. Images are from a single plane.

### Immunoelectron microscopy

Cells were fixed for 24 h at 4 °C in 0.1 M sodium cacodylate buffer (pH 7.4) containing 1% glutaraldehyde and 4% paraformaldehyde, followed by a postfixation with 1% (w/v) osmium tetroxide in the cacodylate buffer for 2 h at 4 °C. The samples were than washed several times in graded series of ethanol and embedded in LR White Resin Grade Acrylic Resin (London Resin Company, Ltd, Aldermaston, UK) at 37 °C 72 h. Ultrathin sections (50 nm thick) were obtained with a MT-2 Sorvall ultramicrotome and collected onto palloidium-coated nickel grid (Electron Microscope Sciences, Hatfield, PA, USA) and then submitted to single immunogold procedure. Briefly, grades carrying ultrathin sections were hydrated with distilled water, followed by rinsing with TBS/glycine (0,02 M, pH 7.2) to avoid crosslinking with the fixative. Nonspecific sites were blocked by incubating the section for 30 min at room temperature (RT) with TBS (pH 7.2) containing 3% BSA, 0.05% NaN_3_ and 0.1% Tween 20, diluted 1 : 1 in normal goat serum. Sections were then incubated overnight at 4 °C with anti-Gal1 antibody (Invitrogen). After being rinsed with washing buffer (TBS, pH 7.2, containing 0.1% BSA, 0.05% NaN_3_ and 0.1% Tween 20), sections were incubated for 2h with donkey anti-mouse IgG coupled with 18 nm gold particles (Jackson ImmunoResearch Laboratories, Inc., West Grove, PA, USA) for 2h at RT and washed in washing buffer. Finally, the samples were fixed with 2.5% glutaraldehyde in 0.1 M sodium cacodylate buffer (pH 7.4) for 10 min (RT). Subsequently, the grids were washed in distillated water, stained with uranyl acetate and lead citrate and examined with a JEOL 100 CX II 100KW transmission electron microscope (Jeol, Tokyo, Japan).

### ELISPOT and *in vivo* cytotoxic assay

The frequency of antigen-specific CD8^+^ T cells was evaluated by ELISPOT analysis of IFN-*γ*-secreting cells^39^ and their effector function was measured by the *in vivo* cytotoxic assay.^17^ Concisely, mice were immunized intramuscularly (i.m.) in each tibialis anterior muscle with 50 *μ*l containing 1 × 10^8^ PFU of human type 5 replication-deficient adenoviruses expressing *β*-galactosidase (Ad*β*-gal). Target cells were obtained from syngeneic WT or Fas-deficient *lpr* C57Bl/6 mice and labeled with carboxyfluorescein diacetate succinimidyl diester (CFSE; Molecular Probes) at final concentration of 10 *μ*M (CFSE_high_) or 1 *μ*M (CFSE_low_). The CFSE_high_ population was pulsed for 40 min at 37 °C with 1 *μ*M of the H-2K^b^-restricted ICPMYARV peptide, a representative *β*-gal epitope (*β*-Gal 497–504, Genscript). Seven days after immunization, mice were inoculated by the retro-orbital route with 2 × 10^7^ CFSE_low_ and 2 × 10^7^ CFSE_high_ cells in a total volume of 200 *μ*l of RPMI-1640 without serum. After 20 h, mice were killed according to procedures approved by the Animal Ethic Committee of our Institute and their spleens removed and processed for ELISPOT and flow cytometry (BD FACSCanto II; BD Biosciences, San Jose, CA, USA). The percentage of specific target lysis was determined using the following formula:


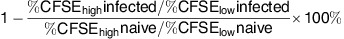


### *In vitro* proliferation and activation profile assay

Lymphocytes were obtained from lymph nodes from WT and *Lgals1*^*−/−*^ C57BL/6 mice and CD8^+^ T cells were enriched using CD8a^+^ T cells Isolation Kit II (Miltenyi Biotec, San Diego, CA, USA) and magnetic cell separation (AutoMACS). The purity of CD8^+^ T cells was >85%. CD8a cells were labeled with 5 mM CFSE and stimulated in 12-well plates for 3 or 6 days with or without 40 U/ml IL-2 and 10 *μ*l/ml, and CD3/CD28 beads (Life Technologies AS, Oslo, Norway). Flow cytometry was performed in a BD FACSVerse (BD Biosciences) and analyzed using FlowJo software (Ashland, OR, USA). The average stage of cell division was calculated based on CFSE histograms gated in the CD3^+^ CD8^+^ T cells.

### Preparation of recombinant Gal1

Purification of recombinant Gal1 was accomplished as outlined previously.^24^ Potential LPS contamination was carefully removed by Detoxi-GelTM (Pierce) and tested using a Gel Clot Limulus Test (<0.5 IU/mg; Cape Cod).

### Co-immunoprecipitation of FasL and Gal1 and flow cytometry

CTLs were isolated from normal spleen (Mouse CD8 Cells Kit, Invitrogen cat. number 11417D) and pre-incubated with or without recombinant Gal1 (25 *μ*g/ml) for 15 min as described.^23^ For co-immunoprecipitation, 500 *μ*g cell lysates were incubated with 2 *μ*g anti-FasL or isotype control antibodies (FasL sc-956; isotype control sc-2027; Santa Cruz Biotechnology, Dallas, TX, USA). The immunocomplexes were captured with Protein G PLUS-Agarose (Santa Cruz Biotechnology) and processed for immunoblotting. Cell surface expression of FasL was analyzed in non-permeabilized cells (2 × 10^5^ cells) by flow cytometry. Briefly, purified CD8^+^ T cells were incubated or not with anti-CD3/anti-CD28 mAb (1 *μ*g/*μ*l clone 145-2C11; 1 *μ*g/*μ*l clone 37.51) for 18 h and then stimulated with PBS or Gal1 for the indicated time periods. Cells were analyzed for FasL expression using the anti-FasL mAb (clone MFL3; eBioscience, San Diego, CA, USA). Nonspecific binding determined with isotype-matched control antibodies is shown. Cells were then analyzed on a FACSAria II flow cytometry (BD Biosciences).

### Degranulation assay (Lamp-1/CD107a externalization)

For CD107a staining, splenocytes collected from WT or *Lgals1*^*−/−*^ C57BL/6 mice were treated with ACK buffer. Staining was performed after *in vitro* culture of splenocytes in the presence or absence of the peptides indicated in each figure. Cells were washed three times in plain RPMI-1640 medium and resuspended in same medium supplemented with 10 mM Hepes, 0.2% sodium bicarbonate, 59 mg/l of penicillin, 133 mg/l of streptomycin, 10% Hyclone fetal bovine serum, 2 mM l-glutamine, 1 mM sodium pyruvate and 55 *μ*M 2-mercaptoethanol. The viability of the cells was evaluated using 0.2% trypan blue exclusion dye to discriminate between live and dead cells. Cell concentration was adjusted to 5 × 10^6^ cells/ml. In half of the cultures, a final concentration of 10 *μ*M of the VNHRFTLV peptide was added. The cells were cultivated in V-bottom 96-well plates (Corning) in a final volume of 200 *μ*l in duplicate, at 37 °C in a humid environment containing 5% CO2. After 8 h incubation, cells were stained for surface markers with CD3 FITC (GK1.5) and CD8 PerCP (53–6.7) and anti-CD107a (1D4B) antibodies on ice for 20 min. At least 8 × 10^5^ cells were acquired on a BD FACSCanto II flow cytometer and then analyzed with FlowJo.

### Statistical analysis

Groups were compared using one way ANOVA followed by Tukey’s HSD test (http://faculty.vassar.edu/lowry/VassarStats.html). The differences were considered significant when the *P*-value was <0.05.

## Figures and Tables

**Table 1:** List of the most abundant proteins identified in 1D SDS-PAGE sample of CTL granule preparation

**Protein ID**	Accession no.	MW/pI	Coverage (%)	Matched peptides
Heat-shock protein HSP90-beta (TSTA)	P11499	83 326/5.0	42	24
NEDD1 protein	P33215	72 970/8.3	23	11
BIP/GRP78	P20029	72 423/5.1	59	38
Heat-shock cognate 71 kDa protein	P08109	70 872/5.4	50	36
Perforin	P10820	62 082/8.4	39	25
T-complex protein 1/TCP-1 theta/CCT-theta	P42932	59 556/5.4	37	17
Zinc finger protein 94 (Zfp-94)	Q9Z1D9	59 156/7.8	35	18
Pyruvate kinase, M2 isozyme	P52480	57 888/7.2	50	31
Protein disulfide isomerase A3 precursor (ERp60)	P27773	56 622/6.0	46	23
Alpha enolase/Enolase-1	P17182	47 141/6.4	55	27
DNAse gamma/DHP2/LS-DNAse/DNAse I-like 3	O55070	35 760/8.9	40	14
Purine nucleoside phosphorylase/Inosine phosphorylase	P23492	32 277/5.8	54	18
Granzyme A precursor/TSP-1/CTLA-3	P11032	28 599/9.5	61	19
Phosphomannomutase 2 (PMM 2)	Q9Z2M7	27 657/6.0	61	15
Granzyme F precursor/MCSP-3/CCP4	P08883	27 643/9.9	40	11
Granzyme E precursor/CCP3	P08884	27 494/9.7	49	13
Granzyme B/CTLA-1/CCP1	P04187	27 470/9.8	50	15
Granzyme G precursor/MCSP-1	P13366	27 381/9.6	61	13
Granzyme C/CCP2	P08882	27 311/9.3	62	17
Ubiquitin carboxyl-terminal hydrolase isozyme L1 (UCH-L1)	Q9R0P9	24 838/5.1	40	10
Ras-related Rab-17	P35292	23 640/5.4	39	7
Ras-related protein Rab-2A	P53994	23 548/6.1	57	9
PPIase precursor/Rotamase/Cyclophilin B	P24369	22 713/9.5	56	15
Peroxiredoxin 1 (thioredoxin peroxidase 2)	P35700	22 177/8.3	41	9
60S ribosomal protein L9	P51410	21 882/10	58	12
Ferritin light chain 1	P29391	20 803/5.7	46	7
Adenine phosphoribosyltransferase/APRT	P08030	19 763/6.3	58	9
Translationally controlled tumor protein (TCTP)	P14701	19 462/4.8	42	12
40S ribosomal protein S10	P09900	18 916/10.2	70	14
Cofilin	P18760	18 560/8.2	69	12
PPIase/Rotamase/Cyclophilin A	P17742	17 972/7.7	45	12
40S ribosomal protein S16	P14131	16 356/10.2	68	11
Superoxide dismutase [Cu-Zn]	P08228	15 943/6.0	54	9
Myosin light chain alkali (MLC3nm)	Q60605	15 731/4.8	62	8
Cystatin C	P21460	15 531/9.2	53	6
Profilin I	P10924	14 957/8.5	46	8
Galectin-1	P10812	14 866/5.3	62	10
Histone H2A	P22752	14 182/10.9	63	7
Histone H2B	P10854	13 936/10.3	65	18

**Table 2:** List of proteins identified by 2D electrophoresis of CTL granule preparation

**Spot**	**Protein ID**	**Accession no**	**MW/pI**	**Coverage (%)**	**Matched peptides**
1	*β*-spectrin/fodrin	Q62261	27 4425/5.7	27	65
2	aBIP/GRP78	P20029	72 423/5.1	54	26
3	aHeat-shock cognate 71 kDa protein	P08109	70 872/5.4	36	20
4	75 kDa glucose regulated protein (GRP 75)/Mortalin	P38647	73 529/5.9	19	8
5	Vimentin	P20152	53 688/5.1	21	12
6	Calreticulin/calregulin/ERP60	NP_031617	48 136/4.33	53	24
7	Calreticulin/calregulin/ERP60	NP_031617	48 136/4.33	54	23
8	aDisulfide isomerase ER-60	P27773	56 622/6.0	42	20
9	Angiopoietin-related protein 2 precursor	Q9R045	57 119/7.3	20	7
10	aAlpha enolase/Enolase-1	P17182	47 453/6.4	76	34
11	aAlpha enolase/Enolase-1	P17182	47 453/6.4	45	18
12	Cathepsin D precursor	P18242	44 954/6.7	34	13
13	Cathepsin D precursor	P18242	44 954/6.7	34	12
14	*β*-actin	NP_001092	42 052/5.29	42	12
15	Aldose reductase	P45376	35 733/6.7	24	7
16	NUDIX	NP_705789	35 623/9.15	38	13
17	aGranzyme E	P08884	27 494/9.7	26	5
18	aGranzyme A/CTLA-3/TSP-1	P11032	28 599/9.5	41	9
19	aGranzyme A/CTLA-3/TSP-1	P11032	28 599/9.5	57	17
20	aGranzyme A/CTLA-3/TSP-1	P11032	28 599/9.5	46	10
21	Chloride intracellular channel protein 1/NCC27/p64 CLCP	Q9Z1Q5	27 013/5.1	44	9
22	Chloride intracellular channel protein 1/NCC27/p64 CLCP	Q9Z1Q5	27 013/5.1	39	9
23	Proteasome subunit alpha type 1/Macropain subunit C2	Q9R1P4	29 547/6.0	34	9
24	Ubiquitin carboxyl-terminal hydrolase isozyme L3 (UCH-L3)	Q9JKB1	26 152/5.0	43	7
25	Phosphoglycerate mutase 1/PGAM-B	Q9DBJ1	28 832/6.7	59	13
26	aGranzyme C/CCP2	P08882	27 311/9.3	75	25
27	Triosephosphate isomerase/TIM	P17751	26 713/6.9	48	13
28	Triosephosphate isomerase/TIM	P17751	26 713/6.9	40	9
29	aTranslationally controlled tumor protein (TCTP)	P14701	19 462/4.8	37	8
30	aTranslationally controlled tumor protein (TCTP)	P14701	19 462/4.8	44	12
31	aAdenine phosphoribosyltransferase/APRT	P08030	19 763/6.3	58	8
32	aGranzyme B/CTLA-1/CCP1	P04187	27 470/9.8	30	7
33	Nucleoside diphosphate kinase B (NDK B)/ NM23-M2	Q01768	17 363/7.0	68	9
34	aPPIase/Rotamase/Cyclophilin A	P17742	17 972/7.7	50	9

a Also identified by 1D electrophoresis.
