# Supplementary figures and images for: Proteomic and functional analysis identifies galectin-1 as a novel regulatory component of the cytotoxic granule machinery
Source: Cell Death Dis. 2017 Dec 7;8(12):e3176–. doi: 10.1038/cddis.2017.506 (PMC5827204; doi:10.1038/cddis.2017.506)

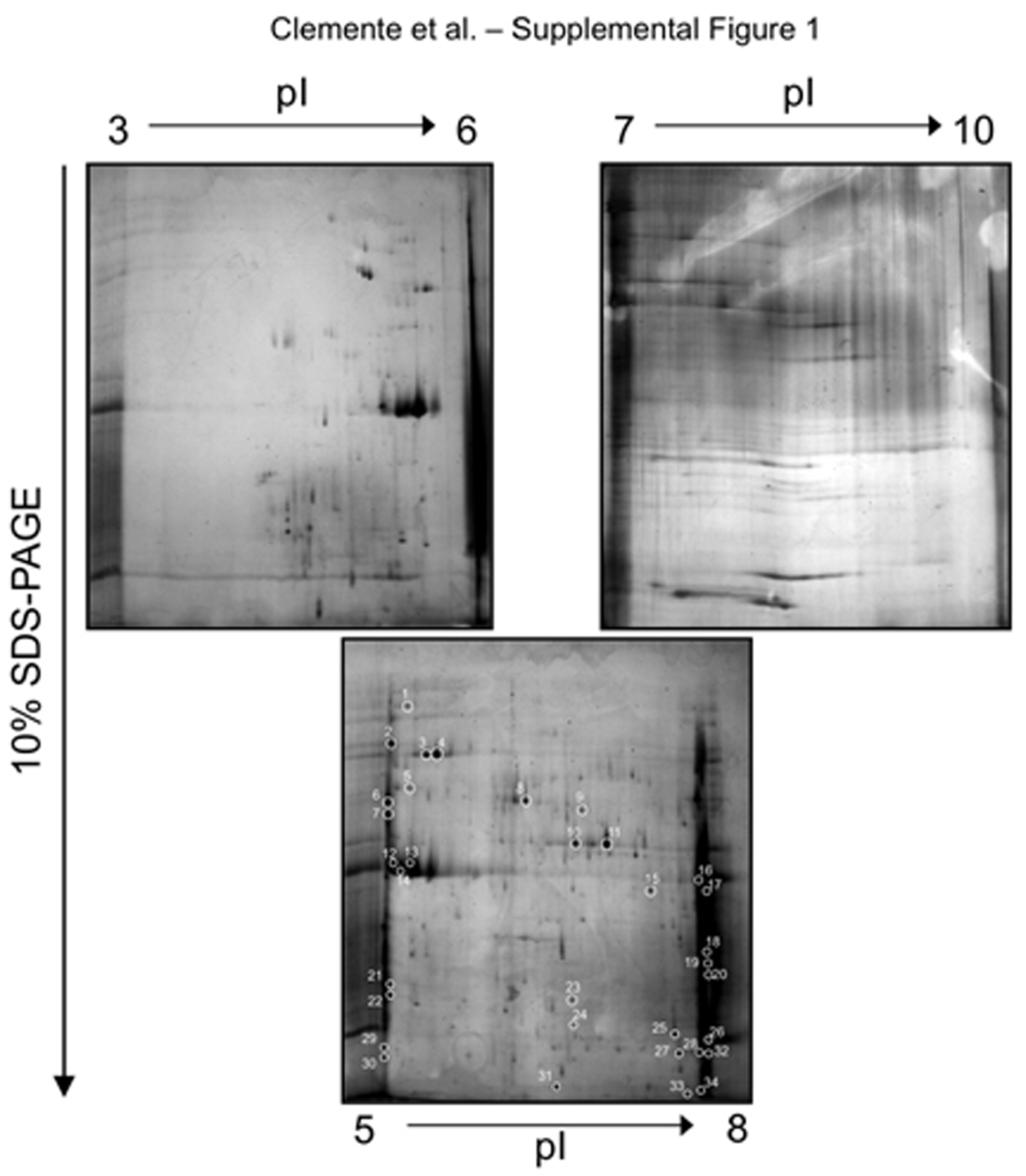

Supplement: Supplementary Figure 1 [file cddis2017506x1.tif]

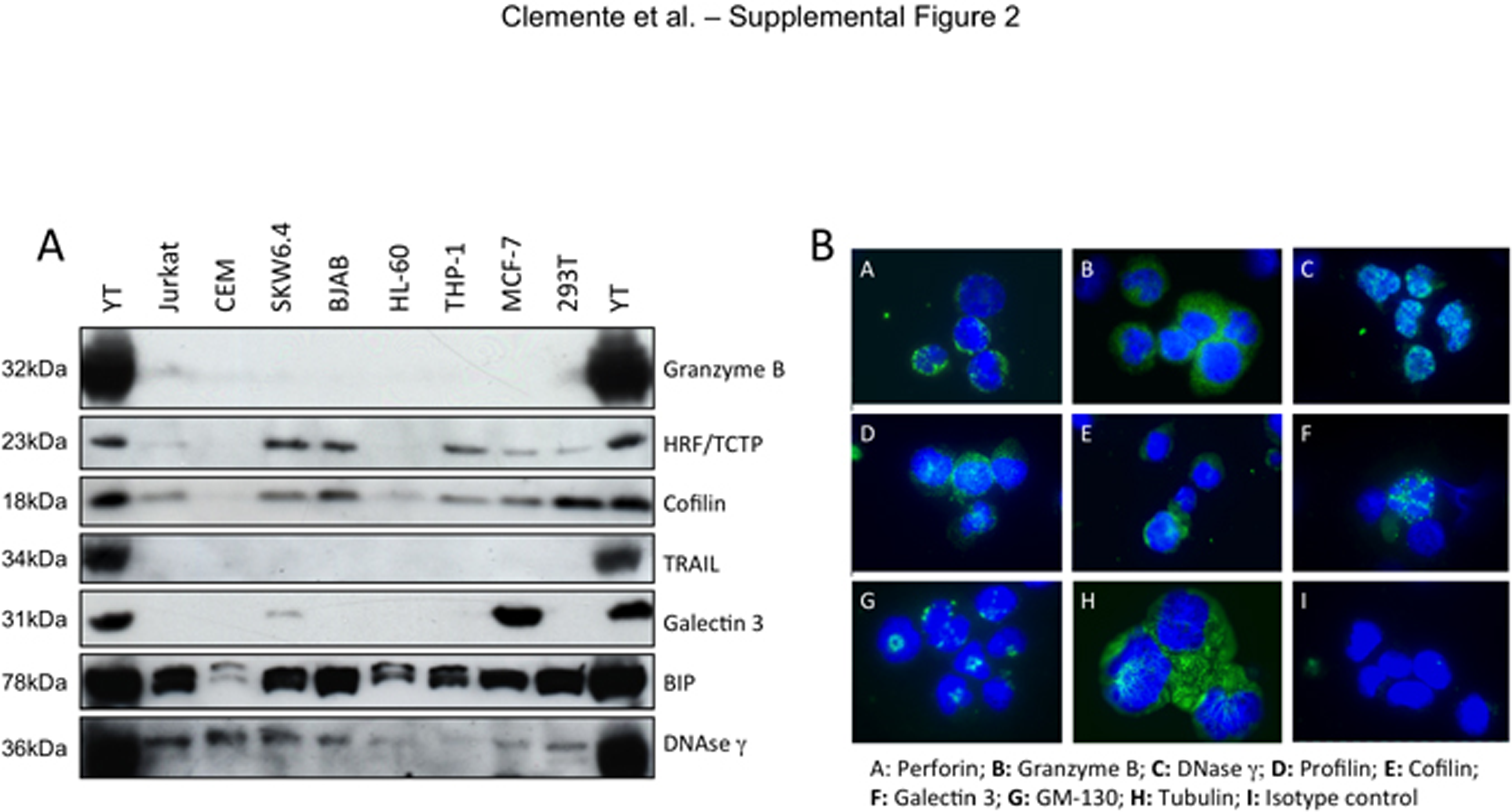

Supplement: Supplementary Figure 2 [file cddis2017506x2.tif]

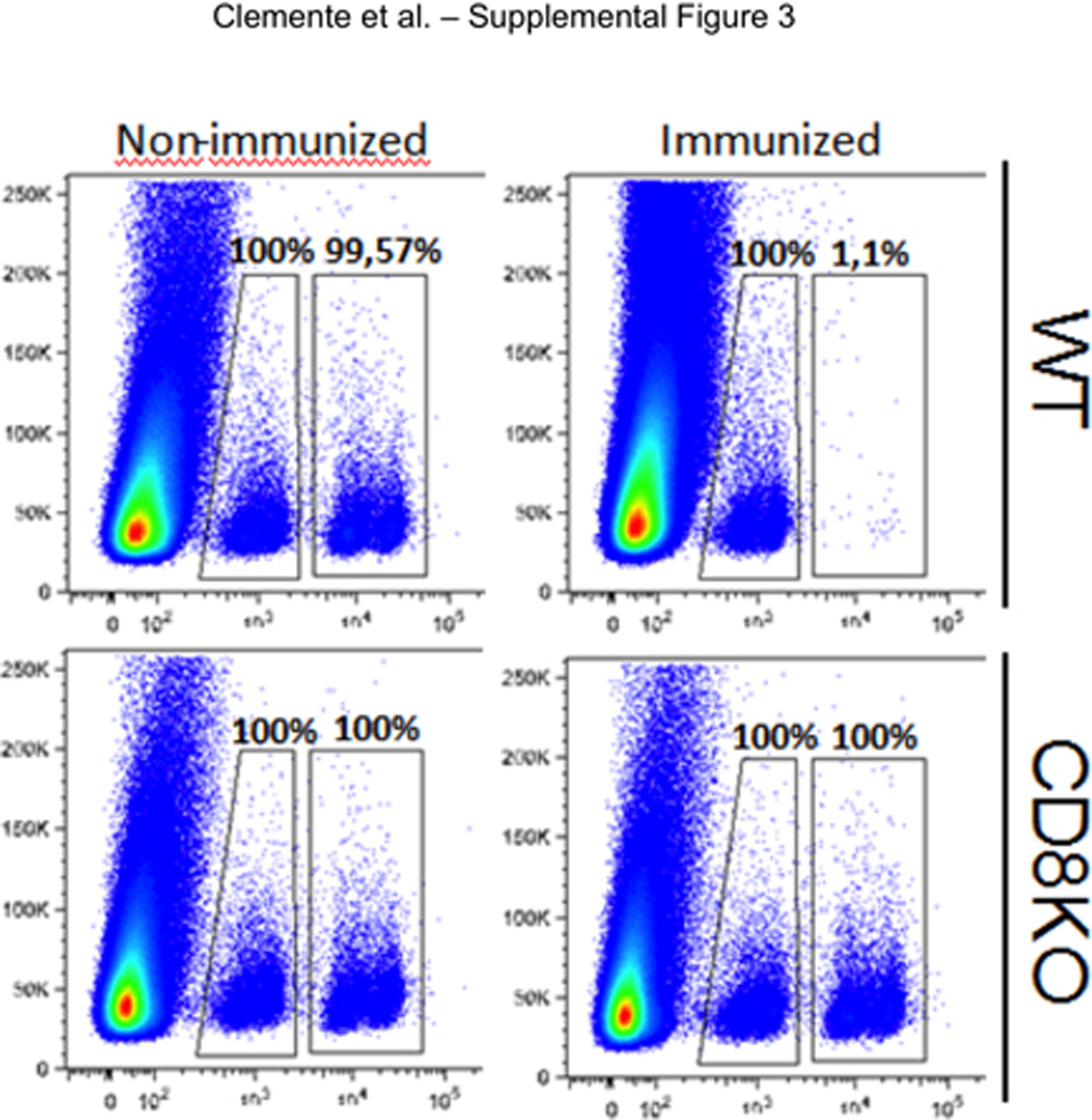

Supplement: Supplementary Figure 3 [file cddis2017506x3.tif]
