# Supplementary material for: Proteomic and functional analysis identifies galectin-1 as a novel regulatory component of the cytotoxic granule machinery
Source: Cell Death Dis. 2017 Dec 7;8(12):e3176–. doi: 10.1038/cddis.2017.506 (PMC5827204; doi:10.1038/cddis.2017.506)
Supplement: Supplementary Figure and Movie Legends [file cddis2017506x6.docx]

**Supplementary Figure and Movie Legends**

**Supplemental Figure 1:** **2D-gel electrophoresis of cytotoxic granules.** Figure shows representative 3-6, 7-10 and 5-8 2D-gel electrophoresis of cytotoxic granules. Labeled spots in gel pI 5-8 depict proteins detected by mass spectometry and displayed in Table II.

**Supplemental Figure 2:** **Validation of mass spec data by Western blot and immunofluorescence. (A)** Western-blot analysis of total cell lysates obtained from different cell lines. **(B)** Immunofluorescence of YT cells showing localization of selected proteins revealed in our proteomic analysis.

**Supplemental Figure 3: The** ***in vivo* cytotoxic assay is dependent on CD8^+^ effector T cells.** WT or CD8^-/-^ C57BL/6 mice were immunized or not with 2 x 10^8^ PFU of Ad5β-gal 8 days before and subjected to *in vivo* cytotoxic assay as described in M&M.

**Supplemental Movies 1 and 2:** 3D reconstruction of Gal1 (Alexa Red 546) and perforin (Alexa 488) distribution in YT cells. Proteins were detected by immunofluorescence and analyzed by confocal microscopy.
