# Supplementary material for: Proteomic and functional analysis identifies galectin-1 as a novel regulatory component of the cytotoxic granule machinery
Source: Cell Death Dis. 2017 Dec 7;8(12):e3176–. doi: 10.1038/cddis.2017.506 (PMC5827204; doi:10.1038/cddis.2017.506)
Supplement: Supplementary Table 1 [file cddis2017506x7.docx]

**Supplemental Table I – List of total proteins identified by mass spectrometry of CTL granule preparation**

| ***Spot*** | ***Protein ID*** | ***MW/pI*** | ***Accession n^o^*** | *Score/Coverage* | *Matched peptides* |
| --- | --- | --- | --- | --- | --- |
| \| 1 \| \| --- \| \| 2 \| \| 3 \| \| 4 \| \| 5 \| \| 6 \| \| 7 \| \| 8 \| \| 9 \| \| 10 \| \| 11 \| \| 12 \| \| 13 \| \| 14 \| \| 15 \| \| 16 \| \| 17 \| \| 18 \| \| 19 \| \| 20 \| \| 21 \| \| 22 \| \| 23 \| \| 24 \| \| 25 \| \| 26 \| \| 27 \| \| 28 \| \| 29 \| \| 30 \| \| 31 \| \| 32 \| \| 33 \| \| 34 \| \| 35 \| \| 36 \| \| 37 \| \| 38 \| \| 39 \| \| 40 \| \| 41 \| \| 42 \| \| 43 \| \| 44 \| \| 45 \| \| 46 \| \| 47 \| \| 48 \| \| 49 \| \| 50 \| \| 51 \| \| 52 \| \| 53 \| \| 54 \| \| 55 \| \| 56 \| \| 57 \| \| 58 \| \| 59 \| \| 60 \| \| 61 \| \| 62 \| \| 63 \| \| 64 \| \| 65 \| \| 66 \| \| 67 \| \| 68 \| \| 69 \| \| 70 \| \| 71 \| \| 72 \| \| 73 \| \| 74 \| \| 75 \| \| 76 \| \| 77 \| \| 78 \| \| 79 \| \| 80 \| \| 81 \| \| 82 \| \| 83 \| \| 84 \| \| 85 \| \| 86 \| \| 87 \| \| 88 \| \| 89 \| \| 90 \| \| 91 \| \| 92 \| \| 93 \| \| 94 \| \| 95 \| \| 96 \| \| 97 \| \| 98 \| \| 99 \| \| 100 \| \| 101 \| \| 102 \| \| 103 \| \| 104 \| \| 105 \| \| 106 \| \| 107 \| \| 108 \| \| 109 \| \| 110 \| \| 111 \| \| 112 \| \| 113 \| \| 114 \| \| 115 \| \| 116 \| \| 117 \| \| 118 \| \| 119 \| \| 120 \| \| 121 \| \| 122 \| \| 123 \| \| 124 \| \| 125 \| \| 126 \| \| 127 \| \| 128 \| \| 129 \| \| 130 \| \| 131 \| \| 132 \| \| 133 \| \| 134 \| \| 135 \| \| 136 \| \| 137 \| \| 138 \| \| 139 \| \| 140 \| \| 141 \| \| 142 \| \| 143 \| \| 144 \| \| 145 \| \| 146 \| \| 147 \| \| 148 \| \| 149 \| \| 150 \| \| 151 \| \| 152 \| \| 153 \| \| 154 \| \| 155 \| \| 156 \| \| 157 \| \| 158 \| \| 159 \| \| 160 \| \| 161 \| \| 162 \| \| 163 \| \| 164 \| \| 165 \| \| 166 \| \| 167 \| \| 168 \| \| 169 \| \| 170 \| \| 171 \| \| 172 \| \| 173 \| \| 174 \| \| 175 \| \| 176 \| \| 177 \| \| 178 \| \| 179 \| \| 180 \| \| 181 \| \| 182 \| \| 183 \| \| 184 \| \| 185 \| \| 186 \| \| 187 \| \| 188 \| \| 189 \| \| 190 \| \| 191 \| \| 192 \| \| 193 \| \| 194 \| \| 195 \| \| 196 \| \| 197 \| \| 198 \| \| 199 \| \| 200 \| \| 201 \| \| 202 \| \| 203 \| \| 204 \| \| 205 \| \| 206 \| \| 207 \| \| 208 \| \| 209 \| \| 210 \| \| 211 \| \| 212 \| \| 213 \| \| 214 \| \| 215 \| \| 216 \| \| 217 \| \| 218 \| \| 219 \| \| 220 \| \| 221 \| \| 222 \| \| 223 \| \| 224 \| \| 225 \| \| 226 \| \| 227 \| \| 228 \| \| 229 \| \| 230 \| \| 231 \| \| 232 \| \| 233 \| \| 234 \| \| 235 \| \| 236 \| \| 237 \| \| 238 \| \| 239 \| \| 240 \| \| 241 \| \| 242 \| \| 243 \| \| 244 \| \| 245 \| \| 246 \| | \| 26S proteasome non-ATPase regulatory subunit 12 \| \| --- \| \| 28S ribosomal proten S15 (MPR-S15)/DC37 \| \| 40s ribosomal protein S10 \| \| 40S ribosomal protein S16 \| \| 60S ribosomal protein L12 \| \| 60S ribosomal protein L7A \| \| 60S ribosomal protein L9 \| \| 75 kDa glucose regulated protein (GRP 75)/Mortalin \| \| Activator of 90 kDa heat shock protein ATPase homolog 1 (AHA1) \| \| Adenine phosphoribosyltransferase (APRT) \| \| Adenomatous polyposis coli protein (APC protein)/mAPC \| \| Adenylate kinase 3 \| \| Adenylate kinase isoenzyme 4 \| \| Aldose reductase \| \| Alpha enolase/Enolase 1 \| \| Alpha-1-acid glycoprotein 1 precursor (AGP 1) \| \| Alpha-internexin \| \| Angiogenin-3 precursor/angiogenin-related protein 2 (EF-5) \| \| Angiopoietin-related protein 2 precursor \| \| Annexin A1/Lipocortin I \| \| Annexin A10 \| \| Annexin A2/Lipocortin II/Calpactin I heavy chain/Chromobindin 8 \| \| Annexin VI/Lipocortin VI/Chromobinding 20/Calphobindin-II (CPB-II) \| \| Annexin VIII \| \| Apopain precursor/CPP32/Yama/Caspase-3 \| \| Ataxia telangiectasia mutated homolog (ATM) \| \| ATP synthase beta chain, mitochondrial precursor \| \| ATP-dependent CLP protease \| \| Baculoviral IAP repeat-containing protein 1a/NAIP \| \| Baculoviral IAP repeat-containing protein 1e/NAIP \| \| BAX, membrane isoform alpha \| \| B-cell lymphoma 6 protein homolog \| \| Beta spectrin \| \| Beta-arrestin 2 \| \| Beta-galactoside alpha-2,3-sialyltransferase/Gal-NAc6S/ST3Gal I \| \| beta-soluble NSF attachment protein (SNAP-beta) \| \| BIP/GRP78 \| \| Breast cancer type 2 susceptibility protein \| \| Bullous pemphigoid antigen 1/Dystonin \| \| Calcitonin gene-related peptide-receptor component protein (CGRP-RCP) \| \| Calcium-binding mitochondrial carrier protein Aralar2/Citrin \| \| Calcium-binding protein CaBP5 \| \| Calcium-binding protein p22/Sid470 \| \| Calneuron 1 \| \| Calsenilin (DRE-antagonist modulator)/DREAM \| \| carbonic anydrase II \| \| Cathepsin D precursor \| \| Chloride intracellular channel protein 1/NCC27/p64 CLCP \| \| Cofilin \| \| Collagen alpha 1 (XII) chain precursor \| \| Collagen alpha 2 (IV) chain precursor \| \| Condensin subunit 1/mCAP-D2/XCAP-D2 homolog \| \| Cystatin C precursor (Cystatin 3) \| \| Cytokeratin 16 \| \| Cytosol aminopeptidase/Leucine aminopeptidase (LAP) \| \| Death effector domain-containing protein (DEDPro1) \| \| Desert hedgehog protein precursor (DHH) (HHG-3) \| \| Developmentally regulated GTP-binding protein 1 (DR \| \| Disulfide isomerase ER-60 \| \| DNA polymerase zeta catalytic subunit/Seizure-related protein 4 \| \| DNA topoisomerase II \| \| DNA-dependent protein kinase catalytic subunit (DNA-PKcs) \| \| DNAse gamma/DHP2/LS-DNAse/DNAse I-like 3 \| \| Dynein heavy chain, cytosolic (DYHC) \| \| Dystrophin  Ecto-5’-nucleotidase/CD73 antigen \| \| eIF4A-II \| \| Elongation factor 1-alpha 1 (EF-1A1)/Elongation factor Tu (EF-Tu) \| \| Epiplakin \| \| Eukaryotic initiation factor 4A-II (eIF4A-II) \| \| Exostosin-like 3 (Multiple exostosis-like protein 3) \| \| Ferritin light chain 1 \| \| fibroblast growth factor receptor 2 precursor (FGFR-2) \| \| FKBP-rapamycin associated protein (FRAP) \| \| Flotillin-1 \| \| Galectin-1 \| \| Galectin-2 \| \| Galectin-3 \| \| Galectin-7 \| \| Glutathione S-transferase Mu 3 \| \| Glutathione S-transferase Yc (GST class-alpha) \| \| Glutathione transferase omega 1 (GSTO 1-1) \| \| Golgi autoantigen (Golgin-160)/MEA-2 \| \| Golgi autoantigen (tGolgin-1) \| \| Golgi coiled coil protein GCC185 \| \| Granzyme A/TSP-1/CTLA-3 \| \| Granzyme B/CTLA-1/CCP1 \| \| Granzyme C/CCP2 \| \| Granzyme E \| \| Granzyme F/MCSP-3/CCP4 \| \| Granzyme G/MCSP-1 \| \| Granzyme K \| \| GRP78/heat shock 70kD protein 5/BiP \| \| GST P2/GST class-pi \| \| GST theta 1/GST class-theta \| \| Guanine nucleotide exchange factor DBS \| \| Heat shock 70kDa protein 12B \| \| Heat shock cognate 71kD protein \| \| Heat-shock-related 70 kDa protein 2 \| \| Heat-shock protein 84 \| \| Heterogeneous nuclear ribonucleoprotein A1 (HDP-1) \| \| Histone H2A \| \| Histone H2B \| \| Homeobox protein HMX2 \| \| Huntingtin/HD protein \| \| Ig-alpha/MB-1 membrane glycoprotein/CD79A \| \| IL-12 alpha chain precursor (IL-12A)/CTL maturation factor 35 kDa (CLMF P35) \| \| IL-17 precursor/Cytotoxic T lymphocyte-associated antigen 8 (CTLA-8) \| \| Interferon-activatable protein 204 (Ifi-204) \| \| Interleukin 1 family member \| \| Jumonji protein \| \| Kelch-like ECH-associated protein 1/Cytosolic inhibitor of Nrf2 \| \| LAF-4 protein/lymphoid nuclear protein related to AF4 \| \| Laminin alpha-3 chain precursor (nicein alpha subunit) \| \| Laminin beta-2 chain precursor/S-laminin (S-LAM) \| \| Lens epithelial cell protein LEP503 \| \| LIM domain kinase 1 (LIMK-1)/KIZ-1 \| \| L-lactate dehydrogenase B chain (LDH-B) \| \| Lymphocyte activation gene-3 protein precursor (LAG-3) \| \| Lysosomal trafficking regulator (beige protein)/CHS1 homolog \| \| MAGUK p55 subfamily member 5/protein associated with Lin-7 1 \| \| Major urinary protein 1 precursor (MUP 1) \| \| MAPK 9/JNK2 \| \| MEK kinase 5 (MEKK 5)/apoptosis signal-regulating kinase 1 (ASK-1) \| \| MEKKK 1/hematopoietic progenitor kinase (HPK) \| \| Metalloproteinase inhibitor 3 precursor (TIMP-3) \| \| Metalloproteinase inhibitor 4 precursor (TIMP-4) \| \| MHC class II associated invariant chain (Ii)/CD74 \| \| Microtubule-actin crosslinking factor 1 (MACF) \| \| Microtubule-associated protein 1B (MAP 1B) \| \| Mitotic spindle assembly checkpoint protein MAD2A \| \| Moesin (membrane-organizing extension spike protein) \| \| Molybdenum cofactor synthesis protein 2 large subunit (MOCS2B) \| \| Myelin P0 protein precursor/Myelin peripheral protein (MPP) \| \| Myeloblastin precursor/proteinase 3 (PR-3) \| \| Myocilin precursor/trabecular meshwork-induced glucocorticoid response protein \| \| Myosin heavy chain (MyHC-alpha) \| \| Myosin IXb (unconventional myosin-9b) \| \| Myosin light chain alkali (MLC3nm) \| \| N-chimaerin (N-chimerin)/alpha chimerin \| \| NEDD1 protein \| \| Nedd-4-like ubiquitin-protein ligase WWP2 \| \| Neurexophilin 1 precursor \| \| Neurobeachin protein (Lysosomal trafficking regulator 2) \| \| Neuronal apoptosis inhibitory protein 2 \| \| NIF3-like protein 1 \| \| Nuclear LIM interactor-interacting factor 1 (NLI-interacting factor)/NIF-like protein \| \| Nuclear receptor co-repressor 2 (N-CoR2)/SMRT/TRAC \| \| Nucleobinding 1 precursor (CALNUC) \| \| Nucleoside diphosphate kinase A (NDK A)/metastasis inhibition factor NM23 \| \| Nucleoside diphosphate kinase B (NDK B)/ NM23-M2 \| \| OX40L \| \| Partitioning defective-6 homolog gamma/PAR6A \| \| Perforin 1 precursor/Cytolysin \| \| Peroxiredoxin 1 (thioredoxin peroxidase 2) \| \| Phosphatidylethanolamine-binding protein (PEBP) \| \| Phosphoglycerate kinase 1 \| \| Phosphomannomutase 1 (PMM 1) \| \| Phosphomannomutase 2 (PMM 2) \| \| Piccolo protein (presynaptic cytomatrix protein)/Aczonin \| \| Plasma glutathione peroxidase precursor (GSHPx-P) \| \| PPIase precursor/Rotamase/Cyclophilin B \| \| PPIase/Rotamase/Cyclophilin A \| \| Probable kinesin light chain 3 (KLC 3) \| \| Probasin precursor (PB) \| \| Profilin I \| \| Prostaglandin F2 receptor negative regulator precursor \| \| Protein disulfide isomerase A3 precursor (ERp60) \| \| Protein disulfide isomerase precursor (PDI) \| \| Protocadherin 15 precursor \| \| Purine nucleoside phosphorylase/Inosine phosphorylase \| \| PYCARD \| \| Pyruvate kinase, M2 isozyme \| \| RAB GDI beta-2/GDI-3 \| \| Rab11 interacting protein Rip11 \| \| Ras-related protein Rab-18 \| \| Ras-related protein Rab-24 \| \| Ras-related protein Rab-27B \| \| Ras-related protein Rab-2A \| \| Ras-related protein Rab-2B \| \| Ras-related protein Rab-5A \| \| Ras-related protein R-Ras \| \| Ras-related Rab-17 \| \| Recoverin/cancer associated retinopathy protein (CAR protein) \| \| Regulator of G-protein signaling 1 (RGS1) \| \| Regulator of G-protein signaling 10 (RGS10) \| \| Retrovirus related POL polyprotein \| \| Rho GDP-dissociation inhibitor 1 (Rho GDI 1) \| \| ribonucleoside-diphosphatase reductase M2 chain \| \| Ribosome-binding protein 1/Ribosome receptor protein (mRRp) \| \| Rod cGMP-specific 3,5-cyclic phosphodiesterase alpha subunit \| \| Sacsin \| \| Salvador homolog 1 protein \| \| Secretagogin \| \| Selenium-binding protein (SN56) \| \| Seprase/Integral membrane serine protease \| \| Serine palmitoyltransferase 2 \| \| Serine/threonine kinase 25/Ste20-like kinase/SOK-1 \| \| Serine/threonine kinase Nek-3/NimA-related protein kinase 3 \| \| Serotransferrin precursor/beta-1-metal binding globulin \| \| Sin3 associated polypeptide p18 \| \| Ski oncogene (C-ski) \| \| Sorting nexin 1 \| \| Spectrin alpha chain \| \| Spectrin beta chain/Fodrin beta chain \| \| Spindlin/SSEC P \| \| Stefin 1 \| \| Structural maintenance of chromosome 2-like 1 protein/XCAP-E homolog \| \| Superoxide dismutase [Cu-Zn] \| \| Synaptonemal complex protein 3 (SCP-3 protein) \| \| Syntaxin 1B \| \| Talin 1 \| \| TBC1 domain family member 1 \| \| T-complex protein 1, theta subunit/TCP-1-theta/CCT-theta \| \| Thiosulfate sulfurtransferase (Rhodanese) \| \| T-lymphoma invasion and metastasis inducing protein 1 (TIAM-1) \| \| TNF receptor associated factor 5 \| \| TRAF-interacting protein \| \| TRAIL \| \| Transcription factor HES-5 \| \| Transgelin 2 \| \| Transgelin/SM22-alpha/Actin-associated protein p27 \| \| Translationally controlled tumor protein (TCTP) \| \| Triosephosphate isomerase/TIM \| \| Tuftelin-interacting protein 11 \| \| Tyrosine kinase LYN \| \| Ubiquitin carboxyl-terminal hydrolase 14/ubiquitin-specific protease 14 \| \| Ubiquitin carboxyl-terminal hydrolase isozyme L1 (UCH-L1) \| \| Ubiquitin carboxyl-terminal hydrolase isozyme L3 (UCH-L3) \| \| Ubiquitin thiolesterase FAF-X \| \| UMP-CMP kinase (cytidylate kinase) \| \| Valyl-tRNA synthetase 2 (VALRS 2) \| \| Vascular endothelial growth factor receptor 1 (VEGFR-1) \| \| Vasodilatador-stimulated phosphoprotein (VASP) \| \| Vesicle-associated membrane protein A (VAMP-A/VAP-A/VAP-33) \| \| Vesomeral secretory protein I precursor (VNSP I)/Lipocalin 3 \| \| Vimentin \| \| Voltage-dependent N-type calcium channel alpha-1B/Brain calcium channel III \| \| WD-repeat protein BING4 \| \| Werner syndrome helicase homolog \| \| X-linked lymphocyte-regulated protein 3A/XLR related protein A12 \| \| Zinc finger protein 198 \| \| Zinc finger protein 35 (Zfp-35) \| \| Zinc finger protein 354A/transcription factor 17 \| \| Zinc finger protein 94 (Zfp-94) \| \| Zinc finger protein HRX (ALL-1) \| \|  \| | \| 52878/6.7 \| \| --- \| \| 29464/10.1 \| \| 18916/10.2 \| \| 13356/10.2 \| \| 17805/9.5 \| \| 29977/10.6 \| \| 21882/10 \| \| 73529/5.9 \| \| 38117/5.4 \| \| 19736/6.3 \| \| 311092/7.4 \| \| 25427/8.9 \| \| 25062/7.0 \| \| 35733/6.7 \| \| 47141/6.4 \| \| 23895/5.6 \| \| 55870/5.2 \| \| 16696/9.1 \| \| 57119/7.3 \| \| 38735/7.0 \| \| 37301/5.4 \| \| 38677/7.6 \| \| 75887/5.3 \| \| 36844/5.6 \| \| 31475/6.5 \| \| 349491/6.5 \| \| 56301/5.2 \| \| 69314/8.1 \| \| 158695/5.9 \| \| 159697/5.6 \| \| 21395/4.9 \| \| 78982/8.3 \| \| 274425/5.7 \| \| 46314/7.6 \| \| 39073/9.3 \| \| 27856/5.2 \| \| 72423/5.1 \| \| 370667/6.2 \| \| 833645/5.2 \| \| 16683/5.0 \| \| 74468/8.8 \| \| 19730/4.4 \| \| 22432/5.0 \| \| 24823/4.8 \| \| 29433/5.5 \| \| 29092/6.5 \| \| 44954/6.7 \| \| 27013/5.1 \| \| 18560/8.2 \| \| 340247/5.5 \| \| 167393/8.8 \| \| 155666/6.0 \| \| 15531/9.2 \| \| 51607/5.1 \| \| 52751/6.6 \| \| 36806/9.2 \| \| 43542/9.5 \| \| 40512/9.0 \| \| 56622/6.0 \| \| 350662/8.6 \| \| 181866/8.5 \| \| 471392/6.7 \| \| 35760/8.9 \| \| 532030/6.0 \| \| 425821/5.7 \| \| 63865/6.2 \| \| 46403/5.3 \| \| 50164/9.1 \| \| 724674/5.7 \| \| 46403/5.3 \| \| 104549/6.2 \| \| 20803/5.7 \| \| 91985/5.5 \| \| 288739/6.7 \| \| 47514/6.7 \| \| 14866/5.3 \| \| 14880/7.0 \| \| 27515/8.5 \| \| 15137/6.7 \| \| 25702/7.6 \| \| 25361/8.8 \| \| 27498/6.9 \| \| 162823/5.3 \| \| 257566/5.3 \| \| 194446/5.1 \| \| 28599/9.5 \| \| 27470/9.8 \| \| 27311/9.3 \| \| 27494/9.7 \| \| 27643/9.9 \| \| 27381/9.6 \| \| 29251/9.8 \| \| 72423/5.1 \| \| 23609/7.7 \| \| 27377/6.8 \| \| 129150/5.6 \| \| 76120/8.8 \| \| 70872/5.4 \| \| 69741/5.6 \| \| 83326/5.0 \| \| 34196/9.3 \| \| 14182/10.9 \| \| 13936/10.3 \| \| 29635/9.2 \| \| 344693/5.9 \| \| 24261/4.8 \| \| 24179/8.4 \| \| 17490/9.2 \| \| 71648/9.0 \| \| 17078/5.7 \| \| 137446/9.5 \| \| 69554/6.0 \| \| 22434/7.7 \| \| 366040/6.3 \| \| 196355/6.3 \| \| 6923/8.6 \| \| 72794/6.5 \| \| 36573/5.7 \| \| 56978/8.5 \| \| 425291/6.1 \| \| 77230/5.7 \| \| 20649/5.0 \| \| 48190/5.6 \| \| 154310/5.5 \| \| 91536/8.1 \| \| 24182/9.0 \| \| 25774/8.2 \| \| 31558/8.6 \| \| 607982/5.4 \| \| 270413/4.8 \| \| 23598/5.2 \| \| 67768/6.2 \| \| 18638/6.3 \| \| 27622/9.5 \| \| 27626/6.7 \| \| 55314/5.5 \| \| 223567/5.6 \| \| 238836/8.8 \| \| 15731/4.8 \| \| 38122/8.6 \| \| 72970/8.3 \| \| 98762/6.7 \| \| 28941/8.9 \| \| 326745/5.8 \| \| 164036/6.1 \| \| 38829/5.9 \| \| 38532/8.8 \| \| 270861/8.5 \| \| 53409/5.0 \| \| 17208/6.8 \| \| 17363/7.0 \| \| 22255/6.1 \| \| 42341/7.4 \| \| 62082/8.4 \| \| 22177/8.3 \| \| 20831/5.2 \| \| 44537/7.5 \| \| 29775/5.3 \| \| 27657/6.0 \| \| 547618/6.1 \| \| 25377/8.3 \| \| 22713/9.5 \| \| 17972/7.7 \| \| 68613/5.8 \| \| 20372/9.5 \| \| 14957/8.5 \| \| 98708/6.0 \| \| 56622/6.0 \| \| 57144/4.8 \| \| 214819/5.0 \| \| 32277/5.8 \| \| 21459/5.3 \| \| 57888/7.2 \| \| 50538/5.9 \| \| 69553/9.2 \| \| 23035/5.2 \| \| 23144/5.9 \| \| 24560/5.4 \| \| 23548/6.1 \| \| 24199/6.2 \| \| 23599/8.3 \| \| 23764/6.3 \| \| 23640/5.4 \| \| 23407/5.1 \| \| 22666/8.8 \| \| 21151/6.4 \| \| 151831/9.7 \| \| 23408/5.1 \| \| 45096/5.3 \| \| 172881/9.4 \| \| 99647/5.4 \| \| 436759/6.7 \| \| 44846/9.1 \| \| 32146/5.1 \| \| 52352/6.0 \| \| 87945/6.6 \| \| 62982/8.4 \| \| 48176/6.3 \| \| 57223/6.4 \| \| 76725/6.9 \| \| 17595/9.4 \| \| 37870/8.1 \| \| 58953/5.1 \| \| 279997/4.9 \| \| 274425/5.7 \| \| 27137/5.3 \| \| 11640/5.9 \| \| 134274/8.5 \| \| 15943/6.0 \| \| 29347/8.7 \| \| 33245/5.3 \| \| 269836/5.8 \| \| 142026/7.0 \| \| 59556/5.4 \| \| 33466/7.7 \| \| 177535/6.2 \| \| 64146/7.7 \| \| 53150/8.9 \| \| 33478/8.2 \| \| 18425/9.7 \| \| 23597/6.6 \| \| 22576/8.9 \| \| 19462/4.8 \| \| 26713/6.9 \| \| 96306/5.6 \| \| 58813/6.7 \| \| 56002/5.1 \| \| 24838/5.1 \| \| 26152/5.0 \| \| 290548/5.7 \| \| 22166/5.7 \| \| 140217/7.9 \| \| 149877/8.6 \| \| 39825/8.7 \| \| 27280/8.6 \| \| 20626/4.6 \| \| 53688/5.1 \| \| 261484/8.9 \| \| 69049/9.8 \| \| 157289/6.1 \| \| 26179/8.9 \| \| 27784/5.2 \| \| 67036/9.0 \| \| 65721/9.5 \| \| 59156/7.8 \| \| 420986/9.2 \| | \| Q9D8W5 \| \| --- \| \| Q9DC71 \| \| P09900 \| \| P14131 \| \| P35979 \| \| P12970 \| \| P51410 \| \| P38647 \| \| Q8BK64 \| \| P08030 \| \| Q61315 \| \| Q9WTP7 \| \| Q9WUR9 \| \| P45376 \| \| P17182 \| \| Q60590 \| \| P46660 \| \| P97802 \| \| Q9R045 \| \| P10107 \| \| Q9QZ10 \| \| P07356 \| \| P14824 \| \| O35640 \| \| P70677 \| \| Q62388 \| \| P56480 \| \| Q9JHS4 \| \| Q9QWK5 \| \| Q9R016 \| \| Q07813 \| \| P41183 \| \| Q62261 \| \| Q91YI4 \| \| P54751 \| \| P28663 \| \| P20029 \| \| P97979 \| \| Q91ZU8 \| \| O35427 \| \| Q9QXX4 \| \| Q9JLK3 \| \| Q62877 \| \| Q9JJG7 \| \| Q9QXT8 \| \| P00920 \| \| P18242 \| \| Q9Z1Q5 \| \| P18760 \| \| Q60847 \| \| P08122 \| \| Q8K2Z4 \| \| P21460 \| \| Q9Z2K1 \| \| Q9CPY7 \| \| Q9Z1L3 \| \| Q61488 \| \| P32233 \| \| P27773 \| \| Q61493 \| \| Q64511 \| \| P97313 \| \| O55070 \| \| Q9JHU4 \| \| P11531 \| \| Q61503 \| \| P10630 \| \| P10126 \| \| Q8R0W0 \| \| P10630 \| \| Q9WVL6 \| \| P29391 \| \| P21803 \| \| Q9JLN9 \| \| O08917 \| \| P10812 \| \| Q9CQW5 \| \| P16110 \| \| O54974 \| \| P19639 \| \| P30115 \| \| O09131 \| \| P55937 \| \| Q91VW5 \| \| Q8CHG3 \| \| P11032 \| \| P04187 \| \| P08882 \| \| P08884 \| \| P08883 \| \| P13366 \| \| O35205 \| \| P20029 \| \| P19157 \| \| Q64471 \| \| Q64096 \| \| Q9CZJ2 \| \| P08109 \| \| P17156 \| \| P11499 \| \| P49312 \| \| P22752 \| \| P10853 \| \| P43687 \| \| P42854 \| \| P11911 \| \| P43431 \| \| Q62386 \| \| P15092 \| \| Q8R459 \| \| Q62315 \| \| Q9Z2X8 \| \| P51827 \| \| Q61789 \| \| Q61292 \| \| Q9WVB6 \| \| P53668 \| \| P16125 \| \| Q61790 \| \| P97412 \| \| Q9JLB2 \| \| P11588 \| \| Q9WTU6 \| \| O35099 \| \| P70218 \| \| P39876 \| \| Q9JHB3 \| \| P04441 \| \| Q9QXZ0 \| \| P14873 \| \| Q9Z1B5 \| \| P26041 \| \| Q9Z223 \| \| P27573 \| \| Q61096 \| \| O70624 \| \| Q02566 \| \| Q9QY06 \| \| Q60605 \| \| Q91V57 \| \| P33215 \| \| Q9DBH0 \| \| Q61200 \| \| Q9EPN1 \| \| Q9QUK4 \| \| Q9EQ80 \| \| P58465 \| \| Q9WU42 \| \| Q02819 \| \| P15532 \| \| Q01768 \| \| P43488 \| \| Q9JK84 \| \| P10820 \| \| P35700 \| \| P70296 \| \| P09411 \| \| O35621 \| \| Q9Z2M7 \| \| Q9QYX7 \| \| P46412 \| \| P24369 \| \| P17742 \| \| Q9DBS5 \| \| O08976 \| \| P10924 \| \| Q9WV91 \| \| P27773 \| \| P09103 \| \| Q99PJ1 \| \| P23492 \| \| Q9EPB4 \| \| P52480 \| \| Q61598 \| \| Q8R361 \| \| P35293 \| \| P35290 \| \| Q99P58 \| \| P53994 \| \| P59279 \| \| Q9CQD1 \| \| P10833 \| \| P35292 \| \| P34057 \| \| Q9JL25 \| \| Q9CQE5 \| \| P11369 \| \| Q99PT1 \| \| P11157 \| \| Q99PL5 \| \| P27664 \| \| Q9JLC8 \| \| Q8VEB2 \| \| Q91WD9 \| \| P17563 \| \| P97321 \| \| P97363 \| \| Q9Z2W1 \| \| Q9R0A5 \| \| Q921I1 \| \| O55128 \| \| Q60698 \| \| Q9WV80 \| \| P08032 \| \| Q62261 \| \| Q61142 \| \| P35173 \| \| Q8CG48 \| \| P08228 \| \| P70281 \| \| P32853 \| \| P26039 \| \| Q60949 \| \| P42932 \| \| P52196 \| \| Q60610 \| \| P70191 \| \| Q8VIG6 \| \| P50592 \| \| P70120 \| \| Q9WVA4 \| \| P37804 \| \| P14701 \| \| P17751 \| \| Q9ERA6 \| \| P25911 \| \| Q9JMA1 \| \| Q9R0P9 \| \| Q9JKB1 \| \| P70398 \| \| Q9DBP5 \| \| Q9Z1Q9 \| \| P35969 \| \| P70460 \| \| Q9WV55 \| \| Q62471 \| \| P20152 \| \| O55017 \| \| Q9Z0H1 \| \| O09053 \| \| Q61806 \| \| Q9CU65 \| \| P15620 \| \| Q61751 \| \| Q9Z1D9 \| \| P55200 \| | \| e5MSFit/38% \| \| --- \| \| e4MSFit/30% \| \| e6MSFit/70% \| \| e5MSFit/68% \| \| e4MSFit/33% \| \| e3MSFit/40% \| \| e6MSFit/58% \| \| e4MSFit/19% \| \| e4MSFit/25% \| \| e5MSFit/58% \| \| e11MSFit/15% \| \| e4MSFit/57% \| \| e4MSFit/39% \| \| e5MSFit/24% \| \| e14MSFit/55% \| \| e4MSFit/36% \| \| e4MSFit/38% \| \| e4MSFit/43% \| \| e5MSFit/20% \| \| e4MSFit/36% \| \| e4MSFit/56% \| \| e4MSFit/28% \| \| e5MSFit/22% \| \| e4MSFit/22% \| \| e5MSFit/43% \| \| e4MSFit/6% \| \| e6MSFit/33% \| \| e4MSFit/25% \| \| e4MSFit/6% \| \| e10MSFit/16% \| \| e4MSFit/40% \| \| e5MSFit/18% \| \| e5MSFit/23% \| \| e4MSFit/20% \| \| e4MSFit/20% \| \| e4MSFit/51% \| \| e15MSFit/54% \| \| e14MSFit/14% \| \| e26MSFit/12% \| \| e4MSFit/36% \| \| e8MSFit/34% \| \| e5MSFit/52% \| \| e4MSFit/43% \| \| e4MSFit/18% \| \| e4MSFit/24% \| \| e4MSFit/28% \| \| e9MSFit/34% \| \| e6MSFit/39% \| \| e6MSFit/71% \| \| e4MSFit/4% \| \| e6MSFit/16% \| \| e4MSFit/11% \| \| e4MSFit/53% \| \| e9MSFit/38% \| \| e5MSFit/34% \| \| e6MSFit/36% \| \| e5MSFit/28% \| \| e5MSFit/32% \| \| e7MSFit/42% \| \| e4MSFit/4% \| \| e6MSFit/20% \| \| e18MSFit/12% \| \| e7MSFit/40% \| \| e14MSFit/13% \| \| e13MSFit/10% \| \| e3MSFit/21% \| \| e5MSFit/29% \| \| e6MSFit/19% \| \| e4MSFit/10% \| \| e5MSFit/22% \| \| e4MSFit/8% \| \| e4MSFit/46% \| \| e4MSFit/14% \| \| e4MSFit/10% \| \| e5MSFit/28% \| \| e4MSFit/62% \| \| e4MSFit/72% \| \| e4MSFit/26% \| \| e5MSFit/67% \| \| e4MSFit/33% \| \| e4MSFit/25% \| \| e5MSFit/58% \| \| e6MSFit/11% \| \| e10MSFit/18% \| \| e5MSFit/13% \| \| e8MSFit/61% \| \| e6MSFit/50% \| \| e14MSFit/75% \| \| e7MSFit/48% \| \| e5MSFit/40% \| \| e5MSFit/61% \| \| e3MSFit/27% \| \| e15MSFit/59% \| \| e4MSFit/54% \| \| e5MSFit/54% \| \| e5MSFit/19% \| \| e5MSFit/23% \| \| MSFit/50% \| \| e4MSFit/29% \| \| e12MSFit/42% \| \| e5MSFit/33% \| \| e4MSFit/63% \| \| e8MSFit/65% \| \| e4MSFit/29% \| \| e10MSFit/11% \| \| e4MSFit/32% \| \| e5MSFit/23% \| \| e4MSFit/50% \| \| e4MSFit/37% \| \| e4MSFit/39% \| \| MSFit/15% \| \| e8MSFit/26% \| \| e7MSFit/56% \| \| e14MSFit/15% \| \| MSFit/14% \| \| e4MSFit/70% \| \| e5MSFit/38% \| \| e4MSFit/17% \| \| e5MSFit/26% \| \| e4MSFit/4% \| \| e5MSFit/20% \| \| e4MSFit/35% \| \| e5MSFit/21% \| \| e4MSFit/14% \| \| e5MSFit/18% \| \| e4MSFit/34% \| \| e4MSFit/37% \| \| MSFit/33% \| \| e27MSFit/17% \| \| e6MSFit/6% \| \| e4MSFit/33% \| \| e5MSFit/43% \| \| e4MSFit/42% \| \| e4MSFit/35% \| \| e4MSFit/42% \| \| e4MSFit/22% \| \| e13MSFit/13% \| \| MSFit/10% \| \| e5MSFit/62% \| \| e5MSFit/37% \| \| e6MSFit/23% \| \| e4MSFit/9% \| \| e4MSFit/42% \| \| e4MSFit/4% \| \| MSFit/15% \| \| e4MSFit/34% \| \| e4MSFit/28% \| \| MSFit/18% \| \| e5MSFit/22% \| \| e4MSFit/53% \| \| e7MSFit/68% \| \| e4MSFit/24% \| \| e4MSFit/14% \| \| e11MSFit/39% \| \| e5MSFit/41% \| \| e5MSFit/55% \| \| e5MSFit/20% \| \| e4MSFit/41% \| \| e7MSFit/61% \| \| e14MSFit/10% \| \| e4MSFit/31% \| \| e8MSFit/56% \| \| e5MSFit/56% \| \| e4MSFit/28% \| \| e4MSFit/39% \| \| e6MSFit/46% \| \| e4MSFit/47% \| \| e10MSFit/46% \| \| e6MSFit/25% \| \| e10MSFit/14% \| \| e8MSFit/54% \| \| e4MSFit/33% \| \| e17MSFit/50% \| \| e5MSFit/27% \| \| e4MSFit/42% \| \| e4MSFit/34% \| \| e5MSFit/35% \| \| e4MSFit/37% \| \| e6MSFit/57% \| \| e4MSFit/36% \| \| e4MSFit/32% \| \| e4MSFit/39% \| \| e4MSFit/39% \| \| e4MSFit/29% \| \| e4MSFit/54% \| \| e4MSFit/41% \| \| MSFit/18% \| \| e5MSFit/23% \| \| e5MSFit/21% \| \| MSFit/26% \| \| e5MSFit/16% \| \| e16MSFit/12% \| \| e5MSFit/31% \| \| e4MSFit/30% \| \| e3MSFit/30% \| \| e5MSFit/16% \| \| e4MSFit/16% \| \| e5MSFit/24% \| \| e8MSFit/35% \| \| e4MSFit/21% \| \| e4MSFit/44% \| \| e5MSFit/31% \| \| e5MSFit/27% \| \| e11MSFit/15% \| \| e8MSFit/27% \| \| e4MSFit/55% \| \| e3MSFit/60% \| \| e5MSFit/20% \| \| e6MSFit/57% \| \| e4MSFit/32% \| \| e4MSFit/31% \| \| e5MSFit/21% \| \| e4MSFit/15% \| \| e6MSFit/31% \| \| e4MSFit/32% \| \| e4MSFit/12% \| \| e4MSFit/24% \| \| e4MSFit/18% \| \| e4MSFit/34% \| \| e4MSFit/44% \| \| e4MSFit/34% \| \| e4MSFit/45% \| \| e7MSFit/44% \| \| e4MSFit/26% \| \| e7MSFit/24% \| \| e5MSFit/26% \| \| e5MSFit/23% \| \| e6MSFit/54% \| \| e5MSFit/43% \| \| e4MSFit/4% \| \| e4MSFit/43% \| \| e5MSFit/30% \| \| e5MSFit/14% \| \| e4MSFit/28% \| \| e4MSFit/13% \| \| e4MSFit/40% \| \| e6MSFit/21% \| \| e5MSFit/10% \| \| e4MSFit/17% \| \| e4MSFit/13% \| \| e4MSFit/51% \| \| e4MSFit/30% \| \| e4MSFit/26% \| \| e8MSFit/34% \| \| e9MSFit/35% \| \| e4MSFit/8% \| | \| 14 \| \| --- \| \| 10 \| \| 14 \| \| 11 \| \| 7 \| \| 12 \| \| 12 \| \| 8 \| \| 6 \| \| 9 \| \| 26 \| \| 10 \| \| 8 \| \| 7 \| \| 27 \| \| 7 \| \| 18 \| \| 6 \| \| 7 \| \| 12 \| \| 9 \| \| 8 \| \| 15 \| \| 8 \| \| 12 \| \| 19 \| \| 13 \| \| 10 \| \| 8 \| \| 18 \| \| 10 \| \| 14 \| \| 49 \| \| 8 \| \| 9 \| \| 7 \| \| 26 \| \| 38 \| \| 54 \| \| 5 \| \| 16 \| \| 9 \| \| 6 \| \| 5 \| \| 9 \| \| 5 \| \| 13 \| \| 9 \| \| 13 \| \| 14 \| \| 18 \| \| 15 \| \| 6 \| \| 16 \| \| 14 \| \| 12 \| \| 8 \| \| 9 \| \| 20 \| \| 17 \| \| 29 \| \| 45 \| \| 14 \| \| 46 \| \| 36 \| \| 9 \| \| 10 \| \| 8 \| \| 17 \| \| 10 \| \| 8 \| \| 7 \| \| 9 \| \| 17 \| \| 10 \| \| 10 \| \| 8 \| \| 7 \| \| 9 \| \| 11 \| \| 8 \| \| 10 \| \| 19 \| \| 32 \| \| 18 \| \| 19 \| \| 15 \| \| 25 \| \| 11 \| \| 11 \| \| 13 \| \| 6 \| \| 38 \| \| 7 \| \| 9 \| \| 18 \| \| 10 \| \| 36 \| \| 15 \| \| 24 \| \| 9 \| \| 7 \| \| 18 \| \| 6 \| \| 24 \| \| 5 \| \| 7 \| \| 6 \| \| 20 \| \| 6 \| \| 20 \| \| 16 \| \| 10 \| \| 38 \| \| 22 \| \| 4 \| \| 9 \| \| 7 \| \| 9 \| \| 13 \| \| 11 \| \| 6 \| \| 9 \| \| 15 \| \| 11 \| \| 10 \| \| 6 \| \| 9 \| \| 63 \| \| 13 \| \| 8 \| \| 23 \| \| 6 \| \| 9 \| \| 4 \| \| 9 \| \| 31 \| \| 21 \| \| 8 \| \| 11 \| \| 11 \| \| 9 \| \| 8 \| \| 12 \| \| 19 \| \| 7 \| \| 8 \| \| 33 \| \| 10 \| \| 6 \| \| 9 \| \| 5 \| \| 4 \| \| 25 \| \| 9 \| \| 7 \| \| 7 \| \| 7 \| \| 15 \| \| 36 \| \| 7 \| \| 15 \| \| 14 \| \| 13 \| \| 8 \| \| 8 \| \| 9 \| \| 23 \| \| 12 \| \| 21 \| \| 18 \| \| 7 \| \| 31 \| \| 9 \| \| 5 \| \| 6 \| \| 10 \| \| 7 \| \| 9 \| \| 7 \| \| 6 \| \| 9 \| \| 7 \| \| 7 \| \| 9 \| \| 8 \| \| 23 \| \| 12 \| \| 9 \| \| 18 \| \| 15 \| \| 41 \| \| 10 \| \| 9 \| \| 6 \| \| 12 \| \| 11 \| \| 10 \| \| 12 \| \| 12 \| \| 5 \| \| 9 \| \| 12 \| \| 23 \| \| 65 \| \| 8 \| \| 6 \| \| 21 \| \| 10 \| \| 10 \| \| 8 \| \| 41 \| \| 15 \| \| 18 \| \| 8 \| \| 14 \| \| 5 \| \| 7 \| \| 8 \| \| 7 \| \| 7 \| \| 8 \| \| 12 \| \| 10 \| \| 18 \| \| 15 \| \| 9 \| \| 10 \| \| 7 \| \| 10 \| \| 10 \| \| 29 \| \| 15 \| \| 9 \| \| 4 \| \| 5 \| \| 12 \| \| 19 \| \| 8 \| \| 16 \| \| 11 \| \| 6 \| \| 12 \| \| 16 \| \| 18 \| \| 24 \| |
